# Intraspecific comparative genomics of isolates of the Norway spruce pathogen (*Heterobasidion parviporum*) and identification of its potential virulence factors

**DOI:** 10.1186/s12864-018-4610-4

**Published:** 2018-03-27

**Authors:** Zhen Zeng, Hui Sun, Eeva J. Vainio, Tommaso Raffaello, Andriy Kovalchuk, Emmanuelle Morin, Sébastien Duplessis, Fred O. Asiegbu

**Affiliations:** 1 Department of Forest Sciences, University of Helsinki, Helsinki, Finland; 2 Collaborative Innovation Center of Sustainable Forestry in Southern China, College of Forestry, Nanjing Forestry University, Nanjing, China; 3 Natural Resources Institute Finland (Luke), Helsinki, Finland; 4 INRA UMR 1136 Interactions Arbres Micro-organismes, INRA Centre Grand Est Nancy, Champenoux, France; 5 UMR 1136 Interactions Arbres/Microorganismes, Faculté des Sciences et Technologies, Université de Lorraine, Vandoeuvre-lès-Nancy, France

**Keywords:** *Heterobasidion parviporum*, Comparative genomics, Virulence factors, Secreted proteins, CpG-biased mutation, Saprotrophic wood decay, Oxidation-reduction process, Transcription factors

## Abstract

**Background:**

*Heterobasidion parviporum* is an economically most important fungal forest pathogen in northern Europe, causing root and butt rot disease of Norway spruce (*Picea abies* (L.) Karst.). The mechanisms underlying the pathogenesis and virulence of this species remain elusive. No reference genome to facilitate functional analysis is available for this species.

**Results:**

To better understand the virulence factor at both phenotypic and genomic level, we characterized 15 *H. parviporum* isolates originating from different locations across Finland for virulence, vegetative growth, sporulation and saprotrophic wood decay. Wood decay capability and latitude of fungal origins exerted interactive effects on their virulence and appeared important for *H. parviporum* virulence. We sequenced the most virulent isolate, the first full genome sequences of *H. parviporum* as a reference genome, and re-sequenced the remaining 14 *H. parviporum* isolates. Genome-wide alignments and intrinsic polymorphism analysis showed that these isolates exhibited overall high genomic similarity with an average of at least 96% nucleotide identity when compared to the reference, yet had remarkable intra-specific level of polymorphism with a bias for CpG to TpG mutations. Reads mapping coverage analysis enabled the classification of all predicted genes into five groups and uncovered two genomic regions exclusively present in the reference with putative contribution to its higher virulence. Genes enriched for copy number variations (deletions and duplications) and nucleotide polymorphism were involved in oxidation-reduction processes and encoding domains relevant to transcription factors. Some secreted protein coding genes based on the genome-wide selection pressure, or the presence of variants were proposed as potential virulence candidates.

**Conclusion:**

Our study reported on the first reference genome sequence for this Norway spruce pathogen (*H. parviporum)*. Comparative genomics analysis gave insight into the overall genomic variation among this fungal species and also facilitated the identification of several secreted protein coding genes as putative virulence factors for the further functional analysis. We also analyzed and identified phenotypic traits potentially linked to its virulence.

**Electronic supplementary material:**

The online version of this article (10.1186/s12864-018-4610-4) contains supplementary material, which is available to authorized users.

## Background

*Heterobasidion parviporum* Niemelä & Korhonen (Basidiomycota; Agaricomycotina; Russulales) is a causative agent of root and butt rot disease of conifers, particularly Norway spruce (*Picea abies* (L.) Karst.). Together with two closely related Eurasian species (*Heterobasidion annosum* (Fr.) Bref. sensu stricto (s.s.) and *Heterobasidion abietinum* Niemelä & Korhonen) and two North American species (*Heterobasidion irregulare* Garbel. & Otrosina and *Heterobasidion occidentale* Otrosina & Garbel.), it constitutes the species complex of *Heterobasidion annosum* sensu lato (s.l.). The annual economic losses due to *Heterobasidion* infection in Europe are estimated at 800 million euros [1]. Due to its huge economic importance, most studies over the past decades have been focused on biology, ecology and control methods of *Heterobasidion* spp. [2]. The mechanisms underlying the pathogenesis and virulence of this species complex remain to be elucidated. This species complex infects stumps and wounds on the roots and stems through basidiospores and spreading to neighboring healthy trees by root-to-root contact [1]. It has dual lifestyles (a saprotroph feeding on wood materials and a necrotroph killing the host tissues and then feeding on dead materials) and the flexible lifestyles interchangeability makes the understanding of the fundamental pathogenesis mechanism challenging. Additionally, the lack of an efficient transformation system in *Heterobasidion* spp. further complicates functional verification studies [2, 3].

Presently, the complete genome sequence is only available for *H. irregulare* TC32–1 among this species complex [4]. Combining quantitative trait locus (QTL) regions with microarray data, 3 candidate genes were proposed to be accountable for the varied virulence between *H. irregulare* and *H. occidentale* [4]. By sequencing 23 *H. annosum* haploid isolates, a genome-wide association study (GWAS) revealed 8 virulence candidate genes located in 7 genomic regions potentially linked to the fungal growth in the sapwood of spruce and pine [5]. Genome comparison between the genotypes of *H. irregulare* and *H. annosum* indicated that pathogenesis-related genes between these two species were more conserved than genes involved in sporulation and saprotrophic decay, implying more significant roles of the latter two traits for the invasiveness of *H. irregulare* [6].

Fungal pathogen genomes driven by their hosts’ evolution are remarkably plastic [7]. Genomic variations could provide insights into the evolutionary forces that shaped the genome architecture and adaptive responses of species of interest [8]. Fungal pathogens produce versatile proteins and small molecules that are subsequently secreted into extracellular spaces in response to changing environment and host conditions [9]. Components of secreted proteins and molecules are functionally relevant in breaking down extracellular carbohydrates and scavenging nutrients [10]. Moreover, phytopathogenic fungi modulate their secretomes to facilitate host colonization, to protect themselves against host-produced reactive oxygen species (ROS), and to subvert host defenses using suites of enzymes including carbohydrate-active enzymes (CAZymes), oxidoreductases, proteases and less well-generalized but often cysteine-rich, small secreted proteins (SSPs) termed effectors [11–13]. Thus, secretome could reflect fungal pathogenicity, virulence and their interactions with hosts.

The absence of *H. parviporum* reference genome results in the scarcity of data about genomic comparison at intra-specific level. Compared to its sibling species, none of the candidate genes potentially associated with its virulence have been hitherto proposed and the relationship of virulence to other fungal fitness-important phenotypic traits remains elusive. Therefore, in this study, we reported on a draft genome of one *H. parviporum* isolate and jointly analyzed this reference with 14 other re-sequenced *H. parviporum* isolates collected from different geographic locations across Finland in order to identify genomic variations that might be relevant for pathogenesis. Exploration of different classes of genes (including core genes, deleted genes, duplicated genes, reference-specific genes, novel genes relative to the reference, genes under strong selection pressure, and genes harboring nonsynonymous single nucleotide polymorphism (SNPs), nonsense mutations or frameshift mutations exclusively found in the least virulent isolate) with secreted protein coding genes more closely scrutinized in each gene class enabled us to propose some virulence candidate genes for functional analysis. Moreover, we characterized the virulence and other phenotypic traits including saprotrophic wood decay, vegetative growth and sporulation of the 15 sequenced isolates. The analysis of the correlations among different traits to virulence shed light on the traits more closely linked to the varied virulence in *H. parviporum* at phenotypic level.

## Methods

### Fungal isolates, single spore isolation and genotyping

All *H. parviporum* isolates of diverse geographic origins were kindly provided by Kari Korhonen (Natural Resources Institute Finland, Luke) and were maintained on 2% malt extract agar plates (MEA). Asexual conidia of each isolate were harvested by flooding the surface of plates with sterile MilliQ water and spread onto new MEA plates. After 24 h of incubation at 22 °C, germinated conidia were picked under microscope (OLYMPUS-CX31), transferred to fresh MEA plates and incubated for 2 weeks. The hyphae of single spore-derived cultures were observed under microscope for clamp connections and genotyped using 4 microsatellite markers (*Ha-ms1, Ha-ms2*, *Ha-ms6* and *Ha-ms10*) as described earlier [14, 15]. Isolates that did not produce clamp connections and contained only one allele at each microsatellite locus analyzed were deemed as homokaryons. The verified homokaryons of each isolate used in this study were deposited at University of Helsinki Fungal Biotechnology Culture Collection (HAMBI/FBCC) (Table 1).

**Table 1:** Summary of *H. parviporum* isolates, mapping and variants relative to the reference isolate S15

Isolate	Name^a^	Origins	HAMBI/FBCC number	Mapped reads	Reads depth (x)	Reads coverage (%)	SNPs	InDels
03020	S1	Pernaja, Horslök	2362	20,338,386	47.76	93.52	146,387	34,661
04121	S2	Artjärvi	2365	20,914,722	49.13	92.97	140,039	34,113
93242	S3	Siilinjärvi	2366	21,352,488	50.13	93.80	138,032	34,749
04051	S4	Mäntsälä	2368	19,057,716	44.78	93.48	136,927	33,259
99055	S5	Kolari, Ylläs	2361	18,024,412	42.34	93.60	144,819	34,027
91271	S6	Karkkila	2363	20,342,018	47.78	95.21	94,638	29,362
99058	S7	Kittilä, Kukasjärvi	2354	20,662,349	48.54	92.44	141,327	32,800
01039	S8	Hattula, Korkee	2369	18,110,965	42.55	93.48	150,875	34,805
96160	S9	Askola	2355	19,847,739	46.58	93.07	135,994	32,959
05029	S10	Kirkkonummi, Yövilä	2364	19,240,310	45.20	93.86	143,212	33,761
99067	S11	Loppi, Launonen	2360	21,492,624	50.51	93.13	143,010	34,019
94174	S12	Loimaa, Köyliön kylä	2358	17,832,237	41.90	92.72	140,559	32,891
98038	S13	Suomusjärvi, Kettula	2370	21,039,687	49.36	93.06	153,255	35,875
03014	S14	Kuhmoinen	2367	19,767,540	46.42	92.79	143,762	33,717
96026	S15	Åland Islands	2359	–	–	–	–	–

^a^Referred name in this study

### Pathogenicity test and phenotypic characterization of fungal isolates

Virulence assays were conducted following a previous study [16]. Briefly, Norway spruce (*Picea abies*) seeds (batch number: R01–00-0902-E461: courtesy of Luke) were surface sterilized with 30% H_2_O_2_ for 15 min, rinsed with sterile MilliQ water and sowed on 1% water agar. Ten germinated seedlings (14–17 days old) were aseptically transferred to a 1% water agar half-covered with moist, sterile filter paper. The root regions of seedlings were inoculated with either 1 ml of homogenized *H. parviporum* mycelia (2–3 weeks old) or sterile MilliQ water as a control and covered with a second moist, sterile filter paper. The parafilm-sealed plates were incubated at 22 °C. Percentage of seedlings killed in each plate was recorded at 15 days post-inoculation (dpi) and 25 dpi with 3 plate replicates at each time point making a combined total of 30 individual seedlings per treatment. Two isolates causing the highest and the lowest mortality rate were further inoculated on 6-year-old Norway spruce clones under greenhouse condition for virulence validation based on induced necrotic lesion length in phloem and xylem (Additional file 1: Method).

Saprotrophic wood decay abilities were tested on heartwood blocks (2 × 1 × 0.5 cm) of Norway spruce according to a previous study [17]. Three wood blocks per treatment pre-dried to constant mass at 65 °C for 24 h were weighed, moisturized in MilliQ water for 1 min and placed in 100 ml flasks containing 1 g of vermiculite (fraction size: 1 mm) and 6 ml of nutrient solution (NH_4_NO_3_ 0.6 g/l, K_2_HPO_4_ 0.4 g/l, KH_2_PO_4_ 0.5 g/l, MgSO_4_.7H_2_O 0.4 g/l, glucose 1.0 g/l). After sealing and autoclaving, flasks were inoculated with either three agar plugs (5х5 mm) from *H. parviporum* isolates pre-grown on Hagem media or sterile Hagem agar plugs as the control, and incubated at 22 °C for 4 months in incubation chambers with humidity maintained at 60–80%. Each treatment has 5 biological replicates. Adhering mycelia were scraped off wood blocks prior to drying at 105 °C for 2 days. Percentage of dry wood mass losses with respect to original dry mass was calculated.

Vegetative growth rate was assayed by deposition of agar plugs (5 х 5 mm) from growing mycelia to the center of 90-mm Hagem agar plates. Radial growth was measured in triplicate at 7 dpi in four perpendicular directions per plate. The average mycelia growth rates (mm/d) were calculated.

After the growth of the isolates for 2 weeks, conidia were dislodged thoroughly from four Hagem agar plugs (7 mm in diameter) by vigorous vortex for 2 min in 10 ml of 0.1% Tween20 solution. Conidial spore concentrations were estimated using a hemocytometer under the microscope (OLYMPUS-CX31). Each isolate has 3 replicates and sporulation was expressed as the number of conidia per milliliter.

### Statistical analysis

After variance homogeneity test, differences among obtained phenotypic traits of the 15 fungal isolates were compared using analysis of variance (ANOVA) followed by Tukey HSD test. Pairwise Pearson correlation coefficients among all traits were calculated. Redundancy analysis was conducted with vegan package [18] to disentangle the contributions of phenotypic traits together with sampling sites of fungal isolates to the variation in the mortality rate of seedlings. Stepwise forward selections based on Akaike Information Criteria (AIC) were used to select the traits that best explained variation in virulence with significance assessed by 999 Monte Carlo permutation tests. Given the reduced model, variation partitioning was applied to quantify the virulence variation attributable to each selected trait, controlling for the influence of the others. All statistical analyses were performed in R v.3.0.2 [19].

### Genomic DNA, RNA extractions and sequencing

Single spore-derived mycelia were harvested from 3-week-old Hagem liquid cultures, grinded in sterile liquid nitrogen-cooled mortars, followed by genomic DNA isolation using DNeasy® Plant Mini Kit (QIAGEN) according to manufacturer’s protocol. Mycelia of the most virulent isolate selected based on virulence assay were used for total RNA extraction using TRI Reagent (Sigma Aldrich) following manufacturer’s instructions. Genomic DNA and total RNA were quantified by NanoDrop spectrophotometer 2000c (Thermo Fisher Scientific Inc.) and qualities were checked via electrophoresis on a 1% agarose gel and Agilent 2100 bioanalyzer, respectively.

A paired-end (PE) (500-bp insert) and a mate-paired (MP) (10-Kb insert) libraries were prepared for the most virulent isolate and sequenced using an Illumina HiSeq 2500 platform (125 bp). An additional 20-Kb PacBio library was constructed and sequenced with a PacBio RSII system (Pacific Biosciences). PE libraries of 300-bp insert (90 bp) were used for remaining fungal isolates and sequenced with an Illumina HiSeq 2500 platform. RNA-seq was conducted with 100-bp PE sequencing using an Illumina HiSeq 4000 platform. All library constructions and sequencing were performed at Beijing Genomics Institute (BGI, Shen-Zhen, China).

### *De novo* genome assembly and whole genome alignment

Reads preprocessing and *de novo* genome assembly of the reference isolate with a hybrid strategy were carried out at BGI. Illumina reads with adapter contaminations, or with more than 10% of ambiguous bases or with more than 40% of low quality (Q score < 20) bases were discarded by SOAPnuke v.1.5.2 (developed by GBI). The quality of processed reads was checked with FASTQC v.0.11.2 (http://www.bioinformatics.babraham.ac.uk/projects/fastqc/). PacBio polymerase reads were processed by SMRT® analysis package v.2.3.0 to filter out reads with quality less than 0.8, to remove adapters and to extract subreads with length of at least 1000 bp. The resulting subreads were self-corrected by Falcon Genome Assembly Tool Kit v.0.3.0 and assembled into contigs by Celera Assembler v.8.3 [20] followed by single base corrections using SOAPsnp v.1.05 [21] and SOAPInDel v.1.08 [22] pipelines with HiSeq PE reads. Scaffolding was performed by SSPACE- LongRead [23] using HiSeq MP reads.

Filtered reads of re-sequenced isolates were assembled *de novo* by VelvetOptimiser v.2.2.5 [24]. The optimized k-mer, expected coverage and coverage cutoffs were obtained through iterative process for various k-mer (37 to 67). Gaps were minimized by GapFiller v.1.10 [25] using original reads via 15 iterations (−m 50, −o 3).

The assembled contigs of re-sequenced isolates were aligned to the reference assembly by NUCmer in MUMmer v.3.23 [26] and filtered by delta-filter utility program to keep one-to-one best mapping of reference to query with at least 1000 bp. The alignments were visualized with dotplots generated by mummerplot and summarized by show-coords utility program in MUMmer package.

### Transposable elements (TEs), simple sequence repeats (SSRs), gene prediction and annotation

TEs in reference assembly were predicted using RepeatScout v.1.0.5 [27], Nseg [28], TRF [29] and classified with REPCLASS v.1.0.1 [30] and tblastx against Repbase Update database v.29.8.2016. Full-length long terminal repeat (LTR) retrotransposons were identified by LTRharvest [31] and LTRdigest [32]. All detected classified TEs were soft-masked by RepeatMasker v.4.0.6 (http://www.repeatmasker.org/) (Additional file 2: Method).

SSRs were scanned by SciRoKo v.3.4 [33] with perfect MISA-mode (default setting). SSR motifs were standardized by grouping motifs in different reading frames and corresponding reverse complements together. The distribution of SSRs relative to protein-coding genes (CDS, introns, 200 bp up- and downstream of CDS, 200–500 bp up- and downstream of CDS and > 500 bp up- and downstream of CDS) were inspected with BEDtools v.2.26.0 [34].

Protein-coding genes were identified using the modified fungal genome annotation pipeline as described by previous studies [35, 36]. After removing adapter contaminations, low quality reads (Q score < 20) and rRNA remnants, remaining RNA-seq reads were constructed into transcripts using both de novo and genome-guided methods (jaccard_clip option on) in Trinity v2.3.2 [37]. For the genome-guided method, RNA-seq reads were first aligned to the genome assembly by GSNAP v.2014.7.21 [38] to generate a BAM file as the input. The resulting transcripts from these two modes were combined and aligned to the genome assembly with GMAP v.2014.7.21 [39] incorporated in Program to Assemble Spliced Alignments (PASA) pipeline v.2.0.2 [40] to build a complete set of transcripts. Homologs from predicted proteins of *H. irregulare* TC 32–1 available in Mycocosm at Joint Genome Institute (JGI) portal (http://genome.jgi.doe.gov/Hetan2/Hetan2.home.html) were searched in the soft-masked assembly by Exonerate v.2.2.0 [41] with protein2genome model. GeneMark-ES v.4.21 [42] with self-training algorithm, fungal branch point model was utilized for ab initio gene predictions. All evidences generated above were sent into EVidenceModeler v.1.1.1 [40] to construct weighted consensus gene structures. The weights were set as 1, 5, and 10 for ab initio predictions, homology-based predictions, and transcripts, respectively. A subset of 806 complete gene models with exact exon-intron boundaries as PASA-constructed transcripts and a support from exonerate-based homologs were manually selected for training Augustus v.3.2.2 [43] and SNAP v.2013.11.29 [44]. The trained programs were used to predict genes ab initio with and without evidence from GSNAP mapping, Exonerate protein alignments and identified known repeats. Thereafter, all predicted gene models were combined into consensus gene structures by EVidenceModeler with the weights of 2, 3, 5, and 7 for ab initio gene predictions without and with hints, homology-based predictions and transcripts, respectively. The complete gene set was subjected to PASA to add UTR annotations based on transcript alignments. The completeness of predicted gene set was assessed by Benchmarking Universal Single-Copy Orthologs (BUSCO) analysis using 1438 fungal single-copy ortholog profiles [45].

The predicted protein sequences were functionally annotated with BLAST2GO v.4.0.7 pipeline [46] (default value). Protein sequences were first blastp against the nonredundant protein database in NCBI (threshold E-value ≤1e-5). Hits were used to assign Gene Ontology (GO) terms to each protein sequence. InterProScan v.5.15–55 [47] was performed to search protein sequences against a collection of databases including Gene3D v.5.5.0, HAMAP v.201511.02, PANTHER v.10.0, Pfam v.28.0, PIRSF v.3.01, PRINTS v.42.0, ProDom v.2006.1, ProSitePattern v.20.113, ProSiteProfiles v.20.113, SMART v.6.2, SUPERFAMILY v.1.75, TIGRFAM v.15.0 to identify present domains and motifs. Meanwhile, available corresponding GO terms for each hit were also retrieved and merged to existing GO annotations from BLAST2GO. CAZymes were annotated by dbCAN v.5.0 with HMMER3 (default setting) [48]. The whole genome was visualized with Circos v.0.69.5 [49].

### Secretome prediction

Secreted proteins were identified using SignalP v.4.1 (sensitive mode) [50] for signal peptide prediction, TargetP v.1.1 [51] for subcellular locations and TMHMM v.2.0 [52] for transmembrane (TM) domain detection. Proteins with more than two TM domains or with one TM domain not overlapping with signal peptide (helix of at least 18 amino acids not in the first 60 amino acids) were excluded. The predicted secretome was further searched in MEROPS v.11.0 [53] and PeroxiBase v.2004–2015 [54] with blastp (E-value ≤1e-4) for proteases and peroxidases, respectively. Potential virulence-related proteins were identified by blastp against pathogen-host interaction database (PHI-base) v.4.2 [55] (E-value ≤1e-5).

### Genome mapping, variant calling and genome-wide nucleotide polymorphism analysis

Processed reads of re-sequenced isolates were mapped to the reference assembly by BWA-MEM v.0.7.12 [56]. Duplicates were removed by Picard MarkDuplicates v2.1.1 (http://broadinstitute.github.io/picard/). SNPs and small insertions/deletions (InDels) collectively termed variants were called by Genome Analysis Toolkit (GATK) v.3.6 [57] HaplotypeCaller and GenotypeGVCFs, followed by hard filtering with VariantFiltration (generic filter recommendations of GATK plus DP > 200.0, DP < 10.0). Nucleotides flanking identified SNPs were retrieved by BEDtools. Variants exclusive to each isolate were pinpointed by VCFtools v.0.1.15 [58]. The impacts of variants on different types of reference genomic regions (intergenic regions, exons, introns, 5’UTR, 3’UTR and exon-intron splicing sites) were predicted with SnpEff v.3.1 [59]. Additionally, the 125 PE (500-bp insert) Illumina reads of the reference isolate were also mapped to the genome of *H. irregulare* TC 32–1 by BWA-MEM.

The individual deduplicated BAM files of re-sequenced isolates were merged by SAMtools v.1.2 [60] to calculate genome-wide nucleotide diversity and Tajima’s *D* in non-overlapping 5 Kb windows using PopBam v.3.0 (−m 10, −× 200, −q 40) [61]. Based on the distribution of *D*-value, outlier windows (*D*-value <5th or > 95th percentile) were selected. Genes with at least 80% of their length overlapped in the outlier windows were extracted.

### Gene classification

Mapping coverage of re-sequenced isolates over the reference gene models was calculated with BEDtools. Coverage breadth was expressed as percentage of nucleotides with minimum one read aligned. Genes were defined as core genes when shared by all isolates with minimum coverage breadth of 80%. Remaining genes exhibited deletions including complete deletions (coverage breadth ≤10%) or partial deletions (coverage breadth < 80% but > 10%) in subset of re-sequenced isolates. Genes only present in the reference isolate were called reference-specific genes. Novel genes relative to the reference were identified by de novo assembling unmapped reads using VelvetOptimiser, predicted using reference-trained Augustus and annotated by blastp against NCBI non-redundant protein database (E-value ≤1e-5), BLAST2GO and InterProScan v.5.22–61.0 against Pfam v.30.0. Copy-number gains were analyzed with CNVnator v.0.3.3 [62] using deduplicated BAM files. Regions with putative copy number gains were extracted using a bin size of 80 bp recommended by the software and filtered with *p*-value < 0.05 and duplication level (q_0_) < 0.5. Genes with at least 80% of their length overlapped in the copy number gained regions were considered as duplicated genes. Significant frequency differences in GO terms between selected gene sets and all gene models were performed by Fisher’s exact test with false discovery rate (FDR) < 0.05. Gene conservation was assessed by the number of variants detected above. Defined conserved genes had less than 5 variants/Kb without non-synonymous SNPs and HIGH impact variants annotated by SnpEFF v.3.1 [59], whereas divergent genes possessed at least 40 variants/Kb.

## Results

### Phenotypic characterizations of homokaryotic isolates

**Fig. 1:**
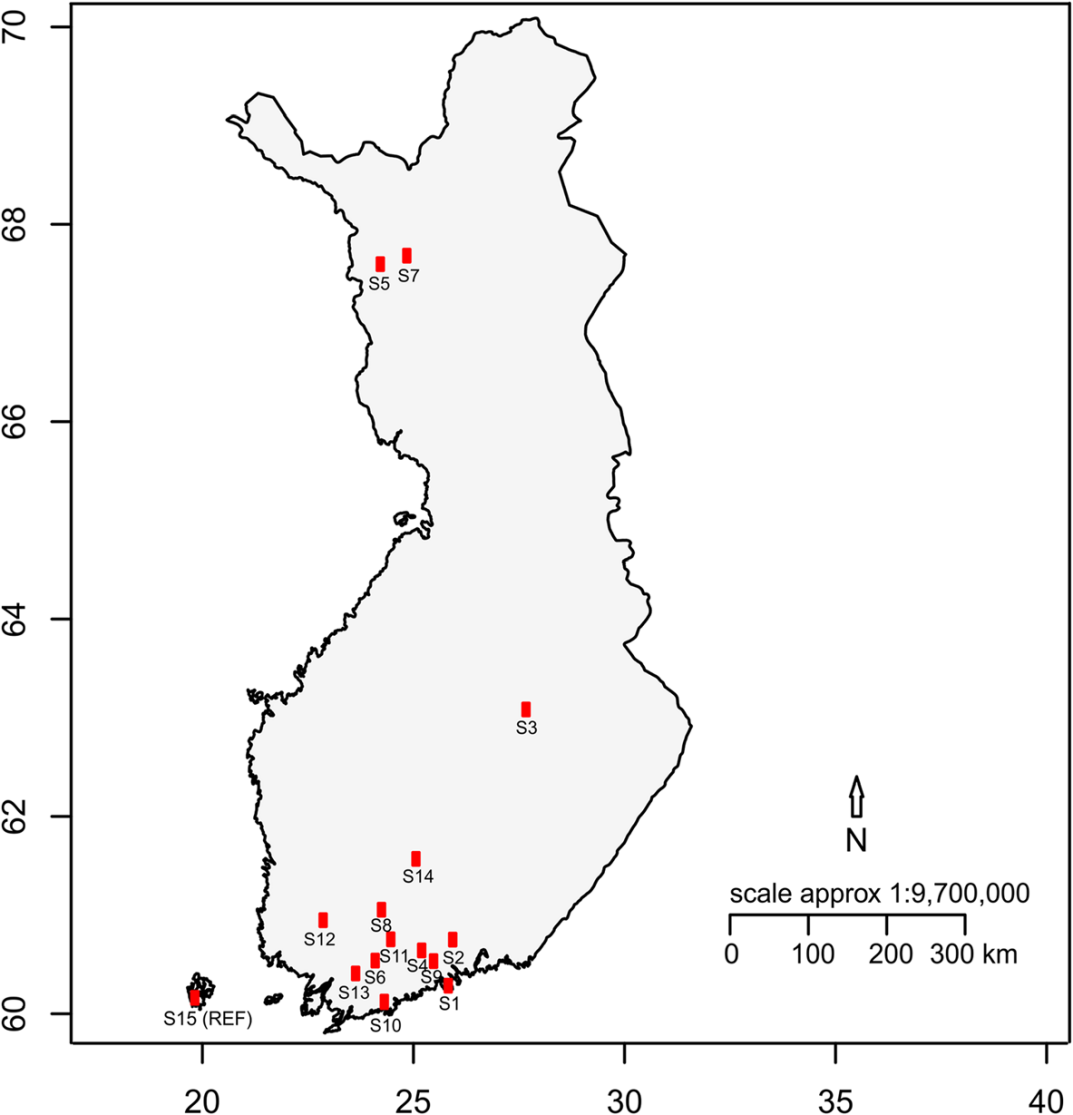
Sampling sites of the 15 *H. parviporum* isolates used in this study in Finland. X- and y-axes are longitude and latitude, respectively

**Fig. 2:**
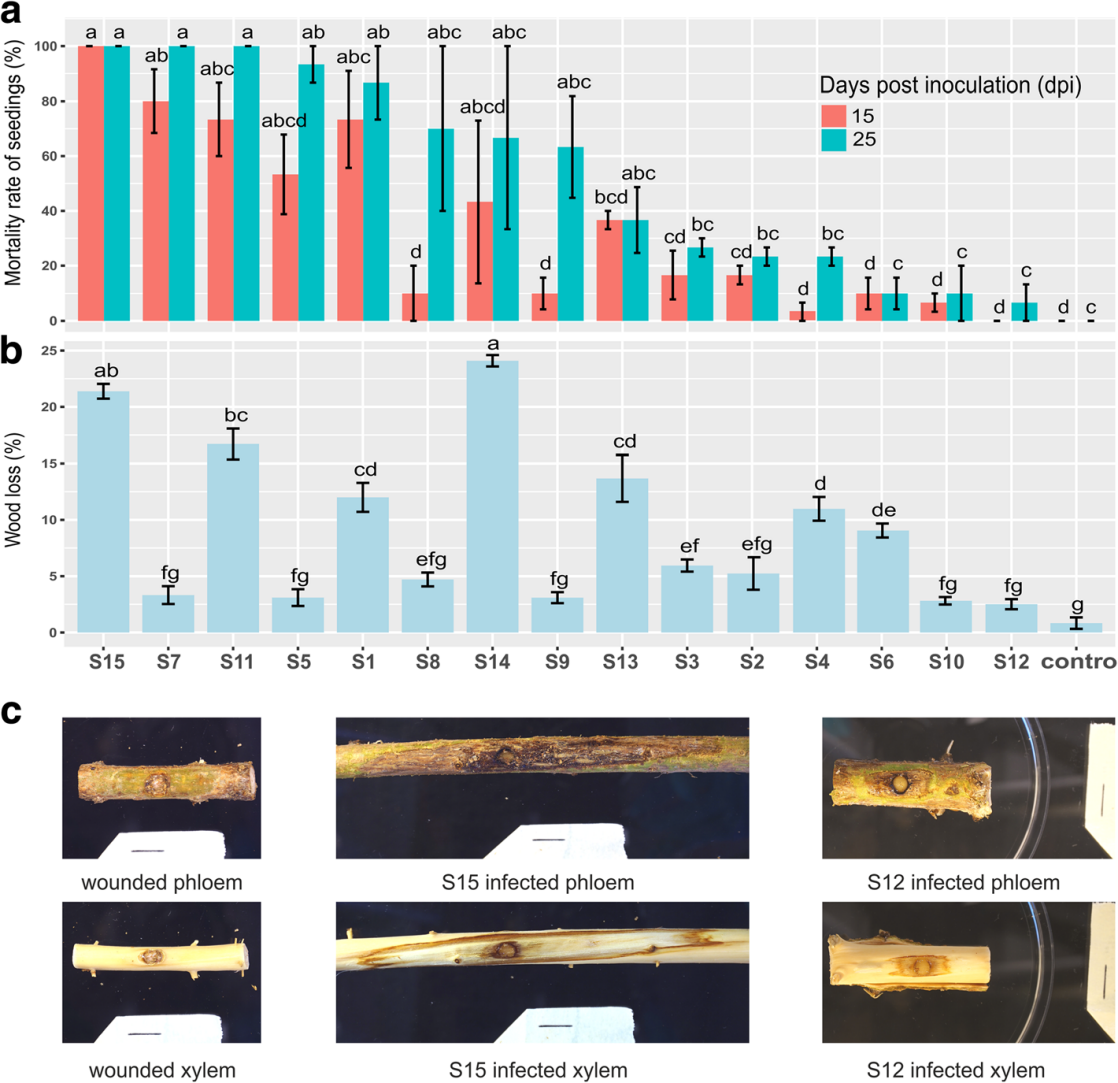
Phenotypic characterization of 15 *H. parviporum* isolates and virulence validation of two selected isolates. **a** Mortality rate of Norway spruce seedlings infected by 15 *Heterobasidion parviporum* isolates and the control at 15 and 25 dpi. **b** Percentage of wood dry mass losses caused by 15 *H. parviporum* isolates and the control. **c** Norway spruce clone C30560 infected by S15 and S12 as well as wounded control in phloem and xylem in virulence validation. Black line is 10 mm scale bar

Inoculation of 15 *H. parviporum* homokaryotic isolates originating from diverse geographic locations across Finland (Table 1, Fig. 1) revealed significant differences in mortality rate of infected spruce seedlings at both 15 dpi (*F* = 8.723, *P* < 0.001) and 25 dpi (*F* = 7.739, *P* < 0.001). Isolate S15 was the most virulent, capable of killing all seedlings at 15 dpi, whereas isolate S12 was the least virulent, resulting in only 6.67% of seedlings killed at 25 dpi (Fig. 2a, Additional file 3: Figure S1). Seedlings in control remained healthy during the experimental period. Variations in wood decay capability, vegetative growth and sporulation were also observed among all isolates (Fig. 2b, Additional file 4: Figure S2 a,b). Further virulence validation of the isolates S15 and S12 in greenhouse using 6-year-old Norway spruce clones supported their drastically differed virulence as reflected by induced necrotic lesion lengths in phloem and xylem (Fig. 2c, Additional file 4: Figure S2c). However, no significant pairwise correlations between the measured traits were observed except for a moderate correlation (*r* = 0.54, *P* < 0.05) between wood decay and mortality rate at 15 dpi. Amongst all measured traits together with longitude and latitude of fungal isolates’ sampling sites, wood decay, latitude and an interaction between these two terms were significant factors in determining the variation in mortality rate, explaining 66.46% of the variance (*P* < 0.01) with latitude (44.46%) being more important than wood decay (17.56%) and their interaction term (16.72%) (Additional file 4: Figure S2d). Interestingly, the significance of latitude depends on the coexistence of wood decay in the initial analytical model and vice versa despite the opposite effects these two factors have on virulence (negative variances in overlapped regions in Venn diagram) (Additional file 4: Figure S2d).

### Genome assembly, transposable elements (TEs), simple sequence repeats (SSRs) and annotation of S15

A total of 30.4 million (PE library) and 15.53 million (MP library) of 125-bp filtered PE reads and 0.42 million of filtered PacBio subreads with average length of 7.3 Kb were assembled into 37.76 Mb, consisted of 287 scaffolds with N50 of 630.6 Kb and GC content of 53% (Table 2). A total of 60.11% reads from PE library of S15 could be properly mapped onto *H. irregulare* TC32–1 genome, covering 66.24% of entire *H. irregulare* genome. This was expectedly lower than *H. annosum* reads coverage on *H. irregulare* genome (75%) as *H. annosum* and *H. irregulare* have more comparable host preference for the genus *Pinus* [6]. The lower genome coverage further necessitated the sequencing of *H. parviporum* reference genome to achieve higher resolution of genetic variations and to facilitate the functional study in this species.

**Table 2:** Summary of genome assembly and annotation in isolate S15

Feature	Value
Scaffold number^a^	287
Total scaffold length (Mb)^a^	37.76
Scaffold N50 (Kb)	630.6
Scaffold N90 (Kb)	89.4
Maximum scaffold length (Mb)	3.49
Minimum scaffold length (Kb)	4.1
GC content (%)^a^	53.00
Number of predicted genes^a^	10,502
Number of genes with RNA-seq support^b^	8859
Mean protein length (aa)	479
Total gene length (Mb)	22.35
Number of genes with Blast hits	9789
Number of genes with InterProScan hits	7941
Number of genes with GO annotations	6977
Benchmarking Universal Single-Copy Orthologs (BUSCOs) assessment^c^	C: 92.6% (1332), D: 11% (158), F: 6.2% (89), M: 1.2% (17)

^a^The corresponding genome information of *H. irregulare* TC32–1 were 15 scaffolds, 33.6 Mb, GC content of 52.23% and 13,405 genes predicted

^b^Based on alignment with constructed transcripts by Trinity v2.3.2 (E-value <1e-5)

^c^C: complete (including complete single copy and duplicated BUSCOs), D: duplicated, F: fragmented, M: missing

**Fig. 3:**
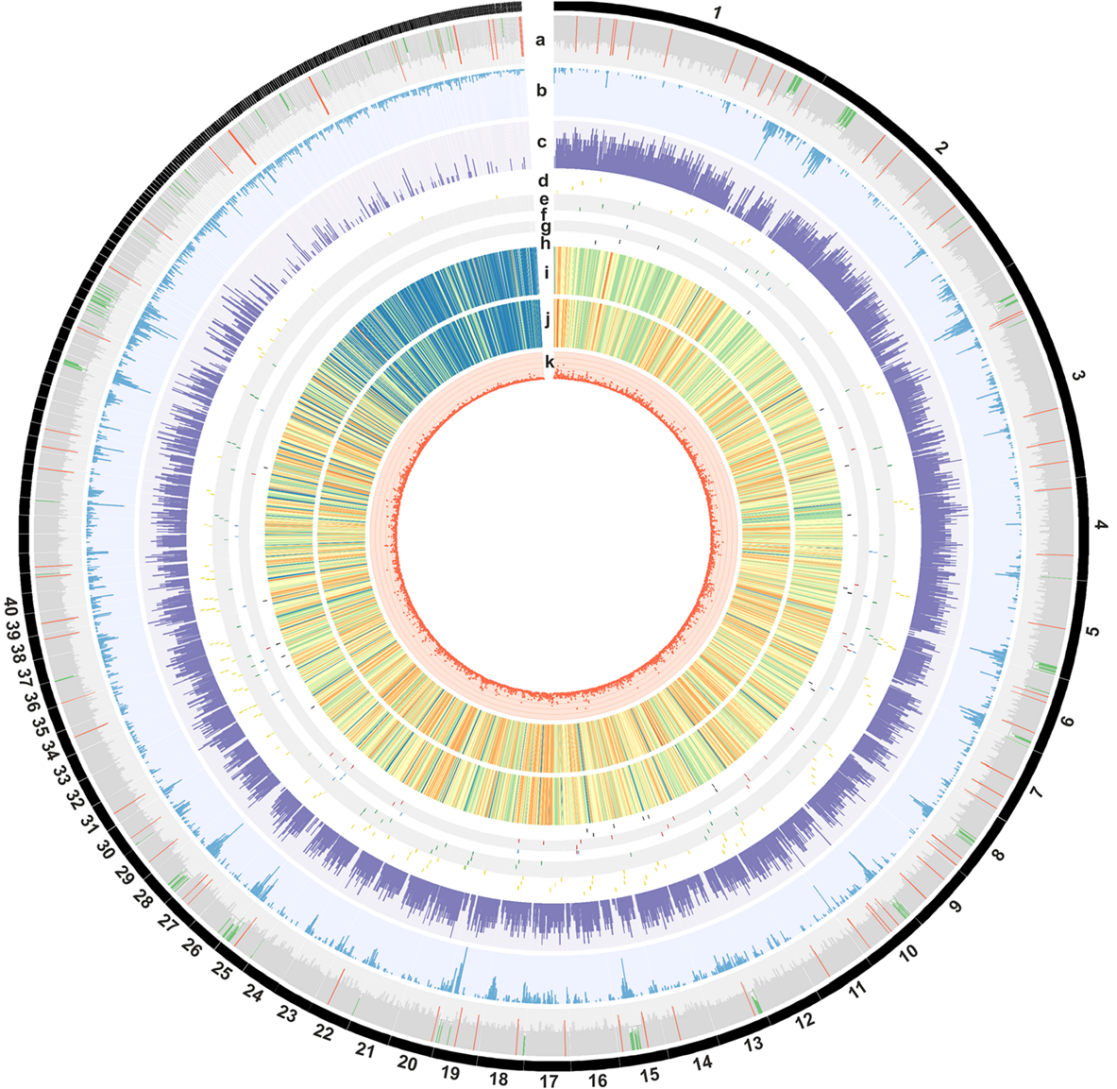
The *Heterobasidion parviporum* S15 genome. The 287 scaffolds were arranged clockwise. The largest 40 scaffolds were labeled. Each circle from the outside to the inside depicts: **(a)** GC content (red > 0.65, green < 0.45); **(b)** Transposable elements density; **(c)** Gene density; **(d)** to **(h)** Secreted CAZymes, peptidase and peptidase inhibitors, peroxidases, cytochrome P450 and effector homologs of PHI-base, respectively; **(i)** Genome-wide nucleotide diversity, color coded from blue to red representing values from low to high; **(j)** SNPs density, color coded from blue to red representing values from low to high; **(k)** InDels density

The identified TEs occupied 20.29% of the assembly, falling into classes of non-LTR retrotransposons (0.28%), LTR retrotransposons (14.68%), DNA transposons (0.92%) and an uncategorized group (5.02%). The most abundant elements were Ty3*/gypsy* retrotransposons, covering 13.36% (5.04 Mb) of the full genome (Additional file 5: Table S1). The genomic regions of low GC content (< 45%) were mainly occupied by TEs (Fig. 3a,b).

The total number of SSRs found was 3066, distributed in 119 scaffolds with average length of 22.90 bp. Trinucleotide SSRs were the most frequent repeat type (Additional file 6: Table S2) and CCG, AGC, ACG were the most abundant SSR motifs (Additional file 7**:** Table S3). When the locations of SSRs were concerned, CDS had the highest number of SSRs (1108) dominated by trinucleotides and hexanucleotides. The pronounced dominance of trinucleotides was also observed in 200 bp and 200–500 bp regions upstream of CDS. In contrast, tetranucleotides were more common in introns, regions 200 bp downstream of CDS and regions located more than 500 bp away from CDS (Additional file 8: Figure S3).

The three classified TE classes were soft-masked in the assembly, from which 10,502 protein-coding genes were predicted and mostly concentrated on larger scaffolds (Table 2, Fig. 3c). In total, 9789 (93.2%) and 7941 genes (75.6%) had blast hits and InterProScan hits, respectively. GO terms could be assigned to 6977 genes (66.44%). A number of 491 genes were categorized into CAZyme families (Additional file 9: Table S4). The BUSCO assessment showed that 1332 (92.6%) out of 1438 nearly universal fungal single-copy orthologs were predicted full-length and 1421 (98.8%) at least partially present with 17 (1.2%) orthologs missing, indicating a high coverage of gene space (Table 2).

### Secretome annotation and PHI-base analysis

In total, 759 putative secreted proteins were predicted, among which 238, 30 and 75 proteins belong to repertoires of secreted CAZymes, peroxidases, peptidases/peptidase inhibitors, respectively (Additional files 10, 11 and 12: Figure S4, Table S5, Table S6). Closer examinations of CAZymes revealed 15, 17, 8, and 16 proteins putatively involved in degradation of plant cell wall components i.e. cellulose, hemicellulose, hemicellulose-pectin complex and pectin, respectively, whereas 43 proteins were predicted to display ligninolytic activities (Additional file 13: Table S7). Additionally, 36 proteins targeting fungal cell wall for exogenous chitin decomposition and recycling of its own cell wall including chitinases (GH18), chitin deacetylase (CE4), glucanases (GH16, GH30, GH55, GH81), and some members of other GH families such as GH27 (α-galactosidases) and GH92 (α-mannosidases) [63] were also present (Additional file 12: Table S6). In brief, S15 possesses a battery of CAZymes for both plant and fungal cell wall degradation and modification with more secreted proteins tailored to lignin degradation. Moreover, 19 proteins such as catalases, haloperoxidases, and thioredoxins required for encoding ROS-scavenging system [64] and putative secreted proteases mainly from Aspartic and Serine families were also found (Additional file 11: Table S5).

To further associate various secreted proteins to fungal virulence, pathogen-host interaction database (PHI-base), containing information on experimentally validated virulence factors in bacteria, fungi and oomycetes, was searched. In total, 211 secreted proteins have hit annotations of “reduced virulence”, “loss of pathogenicity” or “effector_(plant_avirulence_determinant)”, of which 133 proteins were affiliated to CAZymes, peptidases and peroxidases. In particular, aspartic peptidases (A1A), subtilisin-like serine peptidases (S8A), thioredoxins (Trx), ascorbate peroxidases (APx), class II peroxidases, chitinases (GH18) and chitin deacetylase (CE4). Almost all secreted proteins involved in cellulose and lignin degradations were also dominant in the list (Additional files 11, 12 and 13: Table S5, Table S6, Table S7) further substantiating their potential contributions to virulence. Interestingly, all predicted secreted cytochrome P450 (30 proteins) were assigned PHI-base annotations, highlighting the significance of this superfamily in pathogenesis.

### Whole genome comparison

The re-sequenced isolates were assembled de novo and aligned to isolate S15 genome assembly to have a general overview of genomic similarity. The assembly size of isolate S6 was comparable to the reference (39.63 Mb) but with higher degree of fragmentation and larger gaps, whereas the remaining isolates exhibited consistently smaller assembly size (32.76–33.30 Mb) (Table 3). Other properties of all isolates were generally homogenous according to the assembly metrics. After removing alignments of less than 1000 bp, 91–93% of genomic sequences of re-sequenced isolates could be aligned to S15, amounting to 30 Mb with at least 96% sequence identity (Table 3). Notably, S6 had relatively lower percentage of sequences participated in the alignment due to its more fragmented contigs and stringent filtering process. Nonetheless, the aligned regions showed the highest nucleotide identity (Table 3). The matching scaffolds were ordered and all isolates displayed collinear configurations and overall high genomic similarity in comparison to S15 (Additional file 14: Figure S5).

**Table 3:** Summary of assembly of re-sequenced isolates and alignments to S15 genome

Isolates	Contig number	N50 (Kb)	Assembly size (Mb)	GC content (%)	*N* number (%)	Aligned length (Mb)	Aligned sequence (%)	Sequence identity (%)
S1	6116	50.39	33.12	52.87	0.26	30.61	92.42	96.46
S2	5800	55.67	33.02	52.88	0.24	30.21	91.49	96.45
S3	6156	51.20	33.09	52.91	0.21	30.58	92.42	96.60
S4	6328	52.94	33.30	52.89	0.26	30.55	91.75	96.66
S5	6499	49.51	33.19	52.86	0.29	30.49	91.85	96.55
S6	19,952	44.43	39.63	52.96	1.01	30.25	76.33	97.27
S7	6016	45.83	32.93	52.88	0.22	30.25	91.87	96.58
S8	6096	47.81	33.02	52.86	0.23	30.41	92.09	96.52
S9	5838	52.80	32.95	52.88	0.21	30.65	93.01	96.62
S10	5892	52.53	33.16	52.89	0.22	30.68	92.54	96.69
S11	5599	55.60	32.81	52.92	0.20	30.40	92.64	96.59
S12	5883	53.90	32.76	52.94	0.21	30.42	92.85	96.70
S13	6086	47.96	33.28	52.86	0.24	30.39	91.33	96.24
S14	5631	49.64	32.90	52.85	0.19	30.21	91.81	96.43

### Variants identifications, CpG-biased mutations and genome-wide nucleotide polymorphism

Approximately 20 million PE reads of the 14 re-sequenced isolates were mapped onto S15 to assess polymorphism at intra-specific level. The sequencing coverage ranged from 92.44% to 95.21% of the reference at the depth of 42 to 51, resulting in an average of 139,488 SNPs and 33,643 InDels per isolate at 639,222 non-redundant polymorphic sites (Table 1). Among all identified variants of the 14 genomes, only 2.05% (10,747 out of 524,585) of SNPs and 1.07% (1354 out of 126,720) of InDels were shared by all isolates. Therefore, contrary to the SNP density of 3.7/Kb at inter-individual level, a substantially higher density was observed (13.9/Kb) at intra-specific level. Isolate S6 possessed markedly lower number of variants whereas isolate S13 had slightly higher number of variants. This was congruent with sequence identity in the genome alignments which showed that isolate S6 and S13 exhibited the lowest and highest sequence identity respectively (Table 1, Table 3).

Most variants (38.38%) were located in intergenic regions (Additional file 15: Figure S6). The transition to transversion ratio among all SNPs was 3.28 dominated by C-to-T (22.20%) and their complementary bases G-to-A mutations (22.23%). Nucleotides flanking C-to-T changes showed a bias of 40.4% towards G at the 3′ base (i.e. CpG-to-TpG) (Additional file 16: Table S8). As CpG-to-TpG mutation could also increase the occurrences of stop codon (TGA) in open reading frame (ORF) of protein-coding genes, stop codon usages in all predicted genes as well as secreted protein coding genes were checked. As expected, TGA displayed the highest frequency in both gene sets (53.84% and 56.92% respectively), while TAA (19.36%, 20.29%) and TAG (26.80%, 22.79%) showed lower frequencies.

Nucleotide diversity variations and Tajima’s *D* value [65], a measure of the skew of allele frequency distribution across the genomes emphasize the corresponding different evolutionary potentials in different genomic regions. The genome-wide nucleotide diversity was consistent with SNP density (Fig. 3i,j). Tajima’s *D* value assessed in a 5-Kb non-overlapping sliding window manner [66] revealed 6132 windows fulfilling the filtering criteria and the distribution of derived *D* value showed the 5th percentile and 95th percentile to be − 1.9365 and 1.7261 respectively. As positive *D* values represent sequences with an excess of intermediate frequency polymorphism as the result of balancing selection, whereas negative *D* values denote the sequences with a surplus of rare alleles as the consequence of positive selection [67], genes located within positive (379 genes) and negative (393 genes) outlier windows were extracted separately (Additional file 17: Table S9). No enriched GO terms could be revealed in either gene set, probably suggesting different patterns of polymorphism were taking place in response to different classes of genes in need during adaptive evolution. As putative effectors have been shown under positive selection in other fungi [66, 68], secreted protein coding genes located within negative Tajima’s *D* value outlier window were scrutinized (Table 4) and could be prioritized as pathogenesis-related gene candidates for functional analysis following manual gene structure inspection.

**Table 4:** Secreted protein located within outlier window with negative Tajima’s *D* value (*D* < −1.9365)

Gene ID	Brief description	InterPro accession	InterPro name	PHI-hit^a^
evm.scaffold1.1114	CE8	IPR000070; IPR011050	Pectinesterase, catalytic; Pectin lyase fold/virulence factor	–
evm.scaffold13.112	CBM1	IPR000254	Cellulose-binding domain, fungal	Yes^b^
evm.scaffold35.37	CBM50	IPR018392	2 LysM domains	
evm.scaffold67.10	Laccase	IPR001117; IPR011706; IPR011707; IPR008972;	Multicopper oxidase, type 1; Multicopper oxidase, type 2; Multicopper oxidase, type 3; Cupredoxin	Yes
evm.scaffold7.263	AA9	IPR005103	Glycoside hydrolase, family 61	Yes^b^
evm.scaffold15.16	GH10	IPR001000	Glycoside hydrolase family 10 domain;	Yes^b^
evm.scaffold8.166	GH76	IPR005198; IPR008928	Glycoside hydrolase, family 76; Six-hairpin glycosidase-like	–
evm.scaffold7.23	GH92	IPR012939; IPR014718; IPR008928	Glycosyl hydrolase family 92; Glycoside hydrolase-type carbohydrate-binding; Six-hairpin glycosidase-like	–
evm.scaffold38.42	GH92	IPR012939; IPR014718; IPR008928	Glycosyl hydrolase family 92; Glycoside hydrolase-type carbohydrate-binding; Six-hairpin glycosidase-like	–
evm.scaffold2.822	Thioredoxin protein disulfide isomerase (TrxM)	IPR013766	Thioredoxin domain	Yes
evm.scaffold7.192	Chloroperoxidase (HalPrx)	IPR000028	Chloroperoxidase	Yes
evm.scaffold20.57	Phosphatidylethanolamine binding protein (I51)	IPR008914	Phosphatidylethanolamine-binding protein	–
evm.scaffold5.277	Subtilisin-like protease (S08A)	IPR015500; IPR000209; IPR003137; IPR010435	Peptidase S8, subtilisin-related; Peptidase S8/S53 domain; PA domain; Fn3-like domain	–
evm.scaffold53.38	S53 protease (S53)	IPR015366; IPR030400	Peptidase S53, activation domain; Sedolisin domain	–
evm.scaffold53.39	S53 protease (S53)	IPR015366; IPR030400	Peptidase S53, activation domain; Sedolisin domain	–
evm.scaffold16.111	Cytochrome P450	IPR002401	Cytochrome P450, E-class, group I	Yes
evm.scaffold7.193	Phenol 2-monooxygenase	IPR023753; IPR012941;	FAD/NAD(P)-binding domain; Phenol hydroxylase, C-terminal dimerisation domain	–
evm.scaffold10.132	Putative hydrophobic surface-binding protein	IPR021054	Cell wall mannoprotein 1	–
evm.scaffold11.199	Hypothetical protein	IPR006689; IPR005225; IPR027417	Small GTPase superfamily, ARF/SAR type; Small GTP-binding protein domain; P-loop containing nucleoside triphosphate hydrolase	Yes
evm.scaffold14.153	Hypothetical protein	IPR008030; IPR016040	NmrA-like domain; NAD(P)-binding domain	
evm.scaffold27.89	Hypothetical protein	–	–	–
evm.scaffold2.412	Hypothetical protein	IPR000994	Peptidase M24	–
evm.scaffold38.41	Hypothetical protein	IPR001623; IPR011990; IPR019734	DnaJ domain; Tetratricopeptide-like helical domain; Tetratricopeptide repeat	Yes
evm.scaffold4.300	Hypothetical protein	–	–	–
evm.scaffold57.22	Hypothetical protein	IPR017905	ERV/ALR sulfhydryl oxidase domain	–
evm.scaffold58.12	–	–	–	–
evm.scaffold58.13	Fasciclin-like protein	IPR008972	Cupredoxin	–

^a^“Yes” signifies the proteins having hits in PHI-base database and “^b^” means the hits were annotated as effectors

To compare the least virulent isolate S12 more closely with the most virulent reference, secreted protein coding genes that accumulated nonsynonymous SNPs and frameshift mutations exclusively in isolate S12 were collected (Table 5). There was also a point mutation in a third nucleotide position of a codon (TAC to TAA) in one secreted protein, resulting in a premature stop codon. However, this stop gain was circumvented by mutating the original first nucleotide T in that particular codon to C as well (TAC to CAA).

**Table 5:** Secreted proteins exclusively affected by nonsynonymous SNPs and frameshift mutations in S12

Gene ID	Variant type	Brief description	InterPro accession	InterPro name	PHI-hit^a^
evm.scaffold1.948	nonsynonymous SNPs	GH18, CBM5	IPR003610; IPR001579	Carbohydrate-binding module family 5/12; Glycoside hydrolase, chitinase active site	
evm.scaffold3.480	nonsynonymous SNPs	PL1	IPR002022; IPR011050	Pectate lyase Pectin lyase fold/virulence factor	Yes
evm.scaffold67.10	nonsynonymous SNPs	Laccase	IPR001117; IPR011706; IPR011707; IPR008972;	Multicopper oxidase, type 1; Multicopper oxidase, type 2; Multicopper oxidase, type 3; Cupredoxin	Yes
evm.scaffold31.72	nonsynonymous SNPs	Hypothetical protein	–	–	
evm.scaffold35.14	nonsynonymous SNPs	Hypothetical protein	–	–	
evm.scaffold13.87	frameshift mutation	Hypothetical protein	–	–	

^a^“Yes” signifies the proteins cse

### Gene classifications

**Fig. 4:**
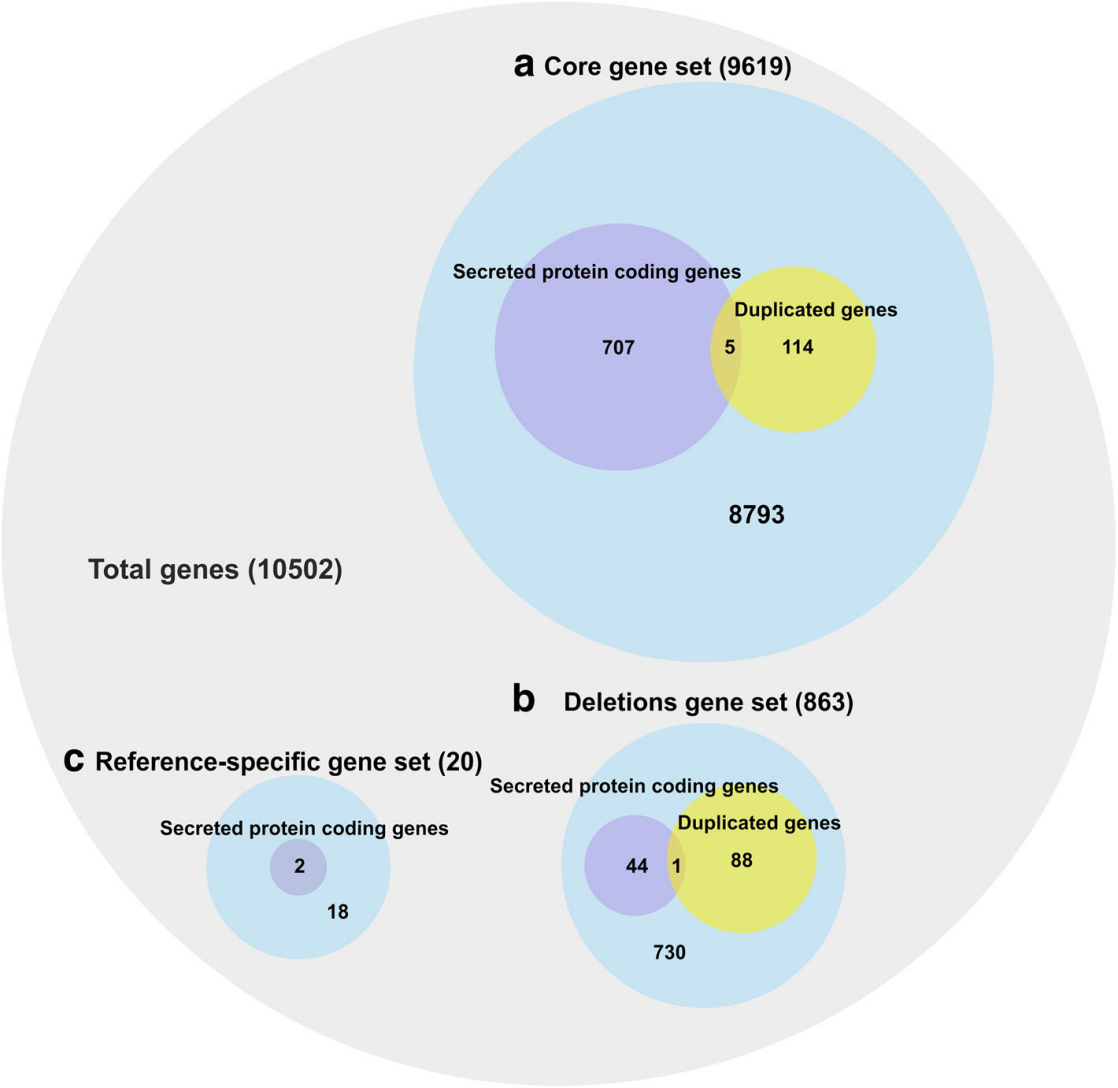
Distribution of gene numbers in each gene category. Gene categories include **(a)** core genes, **(b)** deletion genes, **(c)** reference-specific genes. Duplicated genes and secreted protein coding genes in relation to each type of gene set were displayed

Reads mapping strategy was employed to have a detailed look at how genes were distributed in all isolates under study together with the polymorphisms detected above (Additional file 17: Table S9). In total, 9619 genes were shared by all isolates, whereas 863 genes presented deletions in subsets of isolates. Interestingly, 20 reference-specific genes were also found. A total of 208 genes situated in duplicated regions and from 116 to 190 novel genes were pinpointed. The secreted protein coding genes within each gene set were closely examined (Fig. 4, Additional file 18: Table S10). Isolate S6 harbored the most duplicated and novel genes and the least deleted genes, whereas isolates S3, S12 and S7 had the least duplications, the least novel genes and the most deletions, respectively (Table 6).

**Table 6:** Summary of different categories of genes in relation to S15

Isolates	Duplicated regions^a^	Duplicated genes	Completely deleted genes	Partially deleted genes	Novel genes	Novel genes with Blastp hit	Novel genes with GO terms	Novel genes with domain(s)
S1	62	34	91	212	142	114	39	34
S2	83	52	98	219	153	126	36	34
S3	64	**22**	94	194	134	109	33	34
S4	78	50	59	218	157	124	27	32
S5	67	50	82	195	156	137	35	39
S6	**95**	**54**	**56**	**106**	**190**	**157**	**45**	**42**
S7	81	53	**124**	**229**	155	127	34	34
S8	61	45	107	190	137	111	28	28
S9	**49**	28	95	219	130	104	23	26
S10	71	43	71	197	155	125	30	32
S11	58	32	103	213	121	99	24	24
S12	59	39	106	216	**116**	**95**	**19**	**18**
S13	66	38	84	207	150	116	34	32
S14	80	43	95	219	142	113	27	28

^a^ the largest and the smallest numbers in each category are in bold

The reference-specific genes were mainly located in two genomic regions. Seven genes were on scaffold38 within a fragment of 22.5 Kb, and 9 genes were on scaffold51 occupying a region of 31.4 Kb. There were RNA-seq reads providing support to the existence of those genes. Genome alignments confirmed the absence of these two regions in the 14 isolates, excluding the chances of mis-assembly of reference genome (Additional file 19: Figure S7). Five out of 7 proteins in scaffold38 had hits in PHI-base with either “reduced virulence” or “loss of pathogenicity” annotations (Table 7). On scaffold51, two secreted proteins with no functional annotation and one glucose-6-phosphate isomerase were found (Table 7). One of the secreted proteins (evm.scaffold51.36) had 187 amino acids and was cysteine-rich (20 cysteines), suggesting a likely fungal effector candidate. The remaining genes had no hits in searched databases and were not shown.

**Table 7:** Reference-specific genes on scaffold38 and scaffold51 with annotations

Gene ID	Brief description	GO ID	InterPro accession	InterPro name
evm.scaffold38.25^a^	Short chain dehydrogenase reductase	GO:0016491;GO:0055114	IPR002347; IPR016040	Short-chain dehydrogenase/reductase SDR; NAD(P)-binding domain
evm.scaffold38.27^a^	MFS general substrate transporter	GO:0055085;GO:0016021	IPR011701; IPR020846	Major facilitator superfamily; Major facilitator superfamily domain
evm.scaffold38.28^a^	Alpha beta-hydrolase	–	IPR029058	Alpha/Beta hydrolase fold
evm.scaffold38.29^a^	Cytochrome P450	GO:0005506;GO:0016705;GO:0055114;GO:0020037; GO:0016020	IPR001128	Cytochrome P450
evm.scaffold38.31	Transmembrane	**–**	IPR008547; IPR029058	Protein of unknown function DUF829, TMEM53; Alpha/Beta hydrolase fold
evm.scaffold38.32^a^	Short chain dehydrogenase reductase	GO:0016491;GO:0008152	IPR002347; IPR016040	Short-chain dehydrogenase/reductase SDR; NAD(P)-binding domain
evm.scaffold38.33	Hypothetical protein	GO:0016020	–	Alpha/Beta hydrolase fold
evm.scaffold51.36^b^	–	–	–	–
evm.scaffold51.42	Glucose-6-phosphate isomerase	GO:0004347;GO:0006094;GO:0006096;	IPR001672; IPR035476; IPR035482	Phosphoglucose isomerase (PGI); Phosphoglucose isomerase, SIS domain 1; Phosphoglucose isomerase, SIS domain 2
evm.scaffold51.44^b^	Hypothetical protein	–	–	–

^a^ denotes the proteins having PHI-base database hits

^b^ denotes the secreted proteins

For the core genes, 1457 and 1456 genes fall into highly conserved and divergent gene categories, respectively. There were various significantly enriched GO terms engaged in essential biological processes in the conserved genes (Additional file 20: Table S11), in contrast to the divergent genes, which exhibited no significantly overrepresented GO terms. The most abundant GO term (biological process category) in divergent genes was oxidation-reduction process (GO:0055114) (123 genes) and the most numerous domains were relevant to transcription factors (TFs) such as Zinc finger of C2H2 type, GATA type, and NF-X1 type and Zn(2)-C6 fungal-type DNA-binding domain [69]. Secreted protein coding genes affiliated to the highly conserved genes (89 genes) were significantly associated with main GO terms (assigned to at least 10 sequences) of carbohydrate metabolic process (GO:0005975) and hydrolase activity, hydrolyzing *O*-glycosyl compounds (GO:0004553). Highly divergent secreted proteins (123 genes) displayed significant overrepresentations of main GO terms of oxidation-reduction process (GO:0055114), carbohydrate metabolic process (GO:0005975) and heme binding (GO:0020037) (Additional files 21 and 22: Table S12, Notes). Furthermore, secreted protein coding genes (21.08 variants/kb) were slightly more polymorphic than the overall gene set in terms of variant density (19.59 variants/kb), but opposite result was found in terms of nonsynonymous SNPs density (3.46 nonsynonymous SNPs/kb vs 3.84 nonsynonymous SNPs/kb). Nonetheless, the differences were not significant in either comparison (Mann Whitney test, *P* = 0.9803; permutation t-test, *P* = 0.07449 respectively).

**Fig. 5:**
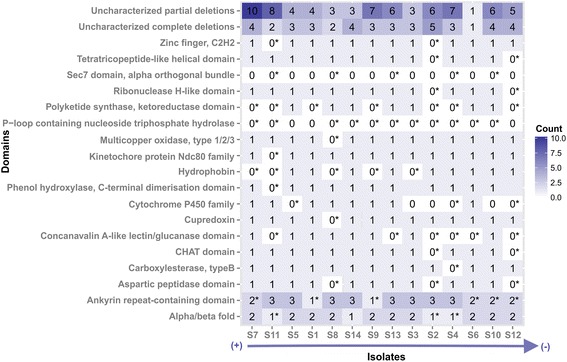
Domain distribution of secreted proteins in the deletion gene set. Symbol “*” signifies the presence of partial deletions but not counted as present in the isolates. Isolates were ranked by their virulence in descending order as in Fig. 2a

To check the gene number variation among the re-sequenced isolates, a gene set with complete or partial deletions in one or more of the isolates was collected. In analogy to the divergent core genes, no significantly enriched GO terms were found. Genes encoding TFs-related domains and those involved in oxidation-reduction process (GO:0055114) were also prevalent. To link putative gene loss (coverage breath ≤0.1) with the weak virulence of isolate S12, the 106 genes completely absent from it were checked. Isolate S7 shared 62 genes, the highest number of complete deletions with S12, despite being the second most aggressive strain on the seedlings (Fig. 2a). There was no single gene that was solely lost in S12 or in the less virulent isolate group S10, S12 and S6. Only 45 secreted proteins (6% of secretome) had deletions. Four secreted proteins i.e. a multicopper oxidase (evm.scaffold14.101), a cytochrome P450 (evm.scaffold10.169), an acid protease (evm.scaffold35.1) and a hypothetic protein (evm.scaffold10.90) had PHI-base hits, putatively contributing to fungal virulence. Interestingly, S12 had partial deletions in 3 of them, whereas isolate S6 being less virulent than most of isolates harbored all of these 4 proteins (Fig. 5).

The 208 genes located within duplicated regions presented GO enrichment of DNA integration (GO: 0015074) (Additional file 21: Table S12). Comparatively larger portion of genes (16 genes) encoded TF-related domains. Three members from cytochrome P450 (evm.scaffold6.188, evm.scaffold6.189, and evm.scaffold18.20) were also found duplicated in one or more of isolates with one (evm.scaffold6.188) to be secreted. There were other 4 secreted proteins with each putatively duplicated in a single isolate in the core genes (Additional file 22: Notes).

**Fig. 6:**
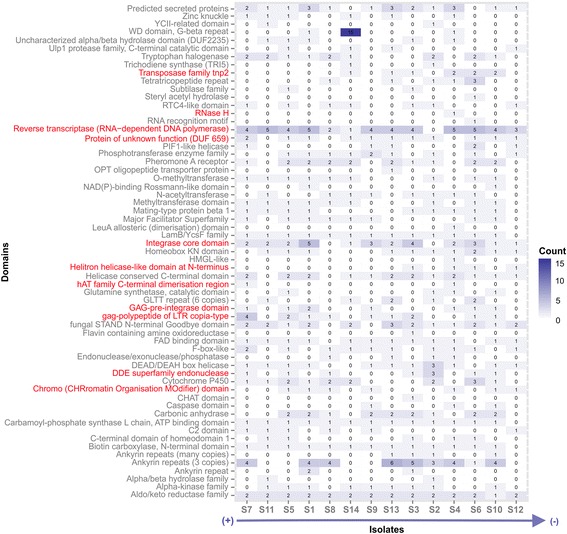
Domain distribution of predicted proteins in the novel gene set. PFAM domains related to TE were highlighted in red. Isolates were ranked by their virulence in descending order as in Fig. 2a

The last group of genes were predicted from unmapped reads and may acquire isolate-specific novel functions. Most predicted genes encoded hypothetical proteins and the most prevalent GO term was nucleic acid binding (GO:0003676) (73 genes) followed by metal ion binding (GO:0046872) and oxidoreductase activity (GO:0016491). The yielded Pfam domains (Fig. 6) included several types of TE-related domains such as reverse transcriptase, integrase and transposases and those involved in gene expression regulations such as zinc knuckle and homeobox KN domain. Additional copies of cytochrome P450 were also found in subset of isolates. A total of 20 secreted proteins were predicted with no hits in searched databases.

## Discussion

Inoculation of homokaryotic *H. parviporum* isolates on Norway spruce seedlings further confirmed that heterokaryosis is not absolutely required for the pathogenicity of *H. parviporum* homokaryons as previously observed [70, 71]. We then examined other phenotypic traits i.e. saprotrophic wood decay, vegetative growth and sporulation important for fungal fitness in *H. parviporum*. No significant correlations could be established between either sporulation or vegetative growth and fungal virulence or wood decay, which might be attributed to the different fungal growth conditions. The use of seedlings in the virulence assay or wood blocks in the decay test might cause isolates to respond differently when compared to axenic culture, where vegetative growth and sporulation were assessed. For example, isolate S15 grew noticeably denser than S12 when infecting seedlings (Additional file 3: Figure S1), illustrating its superiority in colonizing hosts and aggressiveness despite its smaller mycelial growth rate on axenic culture plates. As a root pathogen of coniferous forests, latitude of fungal origins and other environmental factors such as temperature, humidity and soil type might influence the adaptability and aggressiveness of *H. parviporum* isolates. These aspects combined with their different wood decay capability might further affect the fungal virulence. However, this merits further investigation using more number of isolates from wider range of latitude. *H. parviporum* is known to switch between lifestyles (necrotroph versus saprotroph) in response to given environmental conditions. Thriving in one mode (e.g. stronger saprotrophic wood decay ability) might also better guarantee the vitality in the other mode (e.g. higher virulence).

Subsequently, all isolates were subjected to whole genome sequencing to unravel the intra-specific diversity and to explore possible molecular reason(s) underlying the variation in fungal virulence. Repetitive elements have demonstrated roles in genome shaping and adaptive evolution [72]. The observation that Ty3/*Gypsy* are prevailing in *H. parviporum* is in agreement to the to the pattern documented in most published fungal genomes [72]. For SSRs, a pronounced dominance of trinucleotides and hexanucleotides over non-triplet repeats in coding sequences of S15 suggested constraint selections against possible lethal frameshift mutations [73]. The dominances of trinucleotides were also observed in regions 200 bp- and 200–500 bp upstream of CDS, where putative regulatory elements were located, but attenuated with increasing distance to CDS, a similar pattern found in *H. irregulare* [4, 74]. This may reflect a significant role of trinucleotides in the regulation of gene expression with regions closer to CDS more favored by triplet selection process. Furthermore, the over-representation of SSRs in genes enriched in GO terms of transcription factor and regulation of transcription in *H. irregulare* provided yet another evidence of their regulatory functions. Similar to what was observed in *H. irregulare,* tetranucleotides were highly frequent in introns and regions 500 bp away from CDS, indicating less selection over regions more tolerant to reading frame changes. Trinucleotides CCG, AGC and ACG were abundant as similarly observed in *H. irregulare, Ustilago maydis*, and *Fusarium graminearum* [4, 75], suggesting that the processes of generation and fixation of those motifs were not neutral under certain yet to be characterized conditions.

The reads of re-sequenced isolates were both de novo assembled and mapped to the reference genome to inspect the genetic variations at different scales. More than 91% of genomic sequences (except for isolate S6) could be aligned to the reference with at least 96% nucleotide identity across the whole genome. This highlighted an overall high genomic similarity within the *H. parviporum* species, which were further supported by 92–95% of genome coverage by reads. On the contrary, a striking difference of SNP density between inter-individual level (3.7/Kb, when each of isolate was compared to the reference) and intra-specific level (13.9/Kb, when all isolates were considered) reflected a substantial level of polymorphisms. The SNP density of 13.9/Kb in this study was higher than that within its sister taxa *H. irregulare* (4 SNPs/Kb), but lower than that of inter-specific comparison between *H. irregulare* and *H. annosum* (20 SNPs/Kb) [6]. Isolates in this study were collected from Finnish forests of diverse geographic origins, especially with the reference isolate from the secluded Åland Island, which may explain the higher level of polymorphisms than that of *H. irregulare*, which were all from Castelfusano Pinewood Urban Park, Rome [6].

As documented in several studies, high transition to transversion ratio with predominant C-to-T and G-to-A changes might be attributed to repeat-induced point mutation (RIP)-like activities. RIP has been experimentally identified mostly in Ascomycota species such as *Neurospora crassa* [76], *Podospora anserina* [77] and *Leptosphaeria maculans* [78] as one way of fungal-specific genome defense against TEs expansion. Moreover, in the phytopathogen *L. maculans,* RIP could act as a potential mechanism driving the diversification of effector-like genes to boost virulence [79]. In the nematode-trapping Ascomycota fungus *Monacrosporium haptotylum*, RIP has been additionally proposed to enable the generation of novel small-secreted proteins from long secreted proteins via introduction of premature stop codons [80]. *N. crassa* and *L. maculans* had CpA dinucleotide preference at C-to-T mutation sites in TEs [81]. In Basidiomycota, *Microbotryum violaceum, Puccinia graminis, Melampsora larici-populina* and *Rhizoctonia solani* have displayed similar CpG dinucleotide signature as *H. parviporum* presented in this study [81, 82]. However, it remains undetermined whether RIP could target CpG dinucleotides. Alternatively, CpG dinucleotide context preferred by C-to-T changes could have resulted from methylation of cytosine that targeted CpG motifs as a part of epigenetic regulation of gene expression followed by deamination of methylcytosine that is naturally occurring in genomes [83]. Further analysis of C-to-T mutation sites across repetitive elements and protein coding regions in *H. parviporum* is needed to shed more light on the underlying process for this pattern of mutation.

Two reference-specific genomic regions with potential contributions to the fungal virulence were identified. The first region was located in scaffold38, harboring 5 out of 7 genes with PHI-base hits, while the second region was situated in scaffold51 and contained 2 secreted proteins with one of them considered as likely effector candidate. In the divergent core genes, the absence of significantly enriched GO terms suggested that genetic variations have occurred in genes of diverse functions, prevailed by oxidoreductase activity, a similar result when *H. annosum* and *H. irregulare* were compared [6]. Oxidoreductases are implicated in versatile reactions. In our study, the divergent gene family could be exemplified by cytochrome P450. Fungal P450 family has a broad range of functions from the housekeeping process of sterol biosynthesis to the more specialized functions of fungal toxins biosynthesis and xenobiotics detoxification [84, 85]. The diversified functions of this family could be manifested by the overall low sequence similarity except for the conserved domains such as the heme-binding domain or achieved by gene duplication which provides raw evolutionary genetic materials for gene family proliferation and functional diversification [86]. Gene loss is another mechanism to further modulate the P450 family size [85]. In our study, events of P450 gene duplications and gene losses in *H. parviporum* have been documented in their respective gene sets. Additional P450 were further predicted in the novel genes originated from unmapped reads, probably because of their losses from the reference isolate as well. Despite the P450 gene number variation within *H. parviporum* species, no direct relationship to the virulence could be deciphered except for one secreted P450 deleted in the subset of isolates characterized as weak virulence (Fig. 5). In light of these new data, specific classification and function assignments of P450 for *H. parviporum* virulence is needed.

The lack of GO enrichment in the group of genes characterized by deletions indicated that this group included genes with miscellaneous functions. Nonetheless, genes associated with oxidation-reduction process might be more prone to accumulating mutations as well as enduring gene losses as suggested by the abundance of relevant GO terms in both divergent and deletion gene sets. Efforts were then directed to link any particular complete gene deletion in isolate S12 to its weak virulence. Not a single gene was uniquely lost in S12 or in the weak virulence isolate group (S12, S10 and S6) compared to the reference isolate. Intriguingly, isolate S6 exhibited the highest number of genes amongst all isolates, whereas isolate S7 being the second most virulent isolate had the highest number of deletions. Hierarchical clustering of re-sequenced isolates regardless of input data type collected from the deletion set (presence/absence of all genes, GO term counts or secreted proteins domains) could not reveal clustering patterns resembling their virulence profile (data not shown). Due to the redundancy of functions in many gene families such as those common in cell wall degradation [87], it is difficult to find a direct correlation between a particular gene deletion and the fungal virulence. Taken together, it seems most probable that gene loss may not be the main driver for virulence changes.

A remarkable finding across all gene sets was the preponderance of genes encoding transcription factor (TF)-related domains. TFs are crucial players in orchestrating expression of various genes involved in signal transduction [88] and are evolutionarily labile, thereby contributing to phenotypic evolution [89]. In *H. parviporum*, the prevalence of genes encoding TF-related domains in all featured gene sets (divergent, deletion, duplication and novel genes) further reinforced the diversity of evolutionary scenarios that TFs and their associated regulatory networks could possibly be implicated in. For example, C2H2 type of zinc finger protein was found duplicated in almost all isolates, which has been considered as one way of linage-specific expansion for adaptation in vertebrates [88]. It has been documented that deleting genes encoding a secreted superoxide dismutase and catalases could not affect the virulence in the biotrophic pathogen *Claviceps purpurea*, whereas the deletion of a specific TF, ATF1 that functions as a general regulator of catalase activity resulted in an oxidative burst-like reaction in plant tissues and reduced fungal virulence on rye [90, 91]. Since the gene number and the *H. parviporum* virulence were not linear dependent, it is likely that the regulatory networks might be more important than other mechanisms in affecting the fungal virulence by balancing the gene number and robustness of their responses during conifer infections. These were also probably modulated by involvements of mentioned trinucleotides upstream CDS regions or CpG-biased mutation. Therefore, gene expression profiling at specific stages during the host-pathogen interactions may help illuminate the precise role of specific genes. Technically, automated gene annotation may be prone to mistakes and gene number estimates by reads mapping have to be taken with discretion. However, our study was not aimed to obtain the precise gene number of different gene families in all isolates but rather to compare different classes of genes predicted by using the same set of criteria.

Finally, secretome in *H. parviporum* being more polymorphic than the overall gene set but with smaller nonsynonymous SNPs density may reflect the stronger positive selection that are undergoing in the secretome to remove deleterious variants in coding sequences. Therefore, secreted protein coding genes located within outlier window of negative *D* value could be considered as good candidates for future functional analysis.

## Conclusions

We reported on the first reference genome sequence for the Norway spruce pathogen *Heterobasidion parviporum*. Intraspecific comparative genomics analysis revealed the remarkable level of polymorphism in this species with a bias for CpG to TpG mutations, which opens up possibilities of different molecular mechanisms to be uncovered in *Heterobasidion* spp. Two genomic regions exclusively found in the most virulent reference isolate might contribute to its higher virulence. Genes involved in oxidation-reduction process and encoding transcription factor related domains were found to display copy number variation and nucleotide polymorphism in the studied *H. parviporum* isolates. However, not a single gene could be pinpointed as a sole determinant for their varied virulence in this fungal species. This data, therefore, urges the use of RNA-seq to compare the robustness of specific genes during the host infection process. Finally, several secreted protein coding genes proposed based on selection pressure or featured variants could prioritize the selection of putative virulence candidates for further functional analysis.

## Additional files

Additional file 1: Methods. Virulence validation of two selected isolates in greenhouse. (DOCX 12 kb)

Additional file 2: Methods. Transposable elements identification. (DOCX 18 kb)

Additional file 3: **Figure S1.** Norway spruce seedlings infected by isolate S15 (upper row) and S12 (lower row) at 15 dpi and 25 dpi respectively in virulence assay. (TIFF 2918 kb)

Additional file 4: **Figure S2.** (a) Sporulation and (b) Vegetative growth rate of 15 *H. parviporum* isolates. Isolates were sorted by mortality rate at 25 dpi in descending order. (c) Lesion lengths in phloem and xylem caused by isolate S12, isolate S15 and wounding treatments in virulence validation. Different lowercase letters indicate statistically significant differences (*P* < 0.001). Error bars stand for standard errors. (d) Variation partitioned by wood decay, latitude and their interaction term. (TIFF 1303 kb)

Additional file 5: **Table S1.** Summary of transposable elements in S15. (DOCX 14 kb)

Additional file 6: **Table S2.** Summary of simple sequence repeats (SSRs) in S15. (DOCX 12 kb)

Additional file 7: **Table S3.** The most frequent fully standardized SSR motifs in S15. (DOCX 12 kb)

Additional file 8: **Figure S3** Simple sequence repeats (SSRs) distribution in selected genomic regions of S15. Numbers on the bars represent the total SSRs number in that particular type of region. (TIFF 321 kb)

Additional file 9: **Table S4.** Summary of CAZymes in S15 proteome. (DOCX 17 kb)

Additional file 10: **Figure S4** Identified families of peptidase and peptidase inhibitors, peroxidases and CAZymes in isolate S15 secretome. (TIFF 1667 kb)

Additional file 11: **Table S5.** Summary of peptidases, peptidase inhibitors and peroxidases in S15 secretome. (DOCX 14 kb)

Additional file 12: **Table S6.** Summary of CAZymes in S15 secretome. (DOCX 15 kb)

Additional file 13: **Table S7.** Repertoire of CAZymes targeting plant cell walls (PCW) and lignin in S15 secretome. (DOCX 14 kb)

Additional file 14: **Figure S5.** Genome alignment between *H. parviporum* isolate S15 scaffolds and the contigs of re-sequenced isolates. The dot plot represents one to one best mapping, and dots on the diagonal denote co-linearity between the two genomes. Red dots stand for matches in the forward direction and blue dots are inversions relative to S15 scaffolds. (TIFF 2132 kb)

Additional file 15: **Figure S6.** Variant distributions in different type of genomic regions. (TIFF 211 kb)

Additional file 16: **Table S8.** Nucleotides counts and their frequencies within 1 bp flanking C-to-T mutations. (DOCX 13 kb)

Additional file 17: **Table S9.** Annotation of genes in different categories. (a) core genes (b) deleted genes (c) duplicated genes (d) GO terms in novel genes (e) Pfam domains in novel genes (e) genes in negative tajima’s *D* extreme window (EW) (f) genes in positive tajima’s *D* EW. (XLSX 2646 kb)

Additional file 18: **Table S10.** Annotation of secretome in S15 (including all database searches and gene classification results). (XLSX 186 kb)

Additional file 19: **Figure S7.** Dot plots of alignments of S12 contigs to the corresponding regions of reference (a) scaffold38 and (b) scaffold51. (TIFF 301 kb)

Additional file 20: **Table S11.** Significantly over-represented GO terms of conserved core genes compared with all genes in S15. (DOCX 19 kb)

Additional file 21: **Table S12.** Significantly over-represented GO terms of selected test gene sets against all genes in S15. (DOCX 15 kb)

Additional file 22: Notes. Divergent, conserved and duplicated secreted protein coding genes. (DOCX 24 kb)

## Abbreviations

AA: Auxiliary activities
AIC: Akaike Information Criteria
ANOVA: Analysis of variance
APx: Ascorbate peroxidases
BUSCOs: Benchmarking Universal Single-Copy Orthologs
CAZyme: Carbohydrate-active enzyme
CBM: Carbohydrate-binding module
CE: Carbohydrate esterase
DNA: Deoxyribonucleic acid
Dpi: Days post-inoculation
FDR: False discovery rate
GH: Glycoside hydrolase
GO: Gene ontology
GT: Glycosyltransferase
GWAS: Genome-wide association study
InDel: Insertion/deletion
LTR: Long terminal repeat
MEA: Malt extract agar
MP: Mate-paired
PE: Paired-end
PHI-base: Pathogen-host interaction database
PL: Polysaccharide lyase
QTL: Quantitative trait locus
RNA: Ribonucleic acid
ROS: reactive oxygen species
SNP: Single nucleotide polymorphism
SSP: Small secreted protein
SSR: Simple sequence repeat
TE: Transposable element
TF: Transcription factor
TM: Transmembrane
Trx: Thioredoxin
