# Supplementary material for: Intraspecific comparative genomics of isolates of the Norway spruce pathogen (Heterobasidion parviporum) and identification of its potential virulence factors
Source: BMC Genomics. 2018 Mar 27;19:220. doi: 10.1186/s12864-018-4610-4 (PMC5870257; doi:10.1186/s12864-018-4610-4)
Supplement: Supplementary file 1 — Methods. Virulence validation of two selected isolates in greenhouse. (DOCX 12 kb) [file 12864_2018_4610_MOESM1_ESM.docx]

**Methods. Virulence validation of selected isolates under greenhouse conditions**

The tested most virulent and the least virulent isolates of *H. parviporum* were selected to inoculate on three 6-year-old Norway spruce clones (C30560, C30854, and C32539) with 9 plants for each clone under greenhouse condition. Stems were inoculated by 5-mm-diameter agar plugs pre-colonized by actively growing isolates. Two inoculations were made per plant and 3 plants of each clone were inoculated by each isolate and a wounded control, in which the plants were inoculated with sterile agar plugs. A distance of 15 cm was kept between the two inoculation points and between the first inoculations and soil surfaces in the pot. After 6 weeks, the periderm tissues of infected and wounded plants were removed and necrotic lesion length in the phloem was measured. Afterwards, the phloem and vascular cambium were peeled off and lesion length in the xylem was also recorded.
