# Supplementary material for: Intraspecific comparative genomics of isolates of the Norway spruce pathogen (Heterobasidion parviporum) and identification of its potential virulence factors
Source: BMC Genomics. 2018 Mar 27;19:220. doi: 10.1186/s12864-018-4610-4 (PMC5870257; doi:10.1186/s12864-018-4610-4)
Supplement: Supplementary file 2 — Methods. Transposable elements identification. (DOCX 18 kb) [file 12864_2018_4610_MOESM2_ESM.docx]

**Methods. Transposable elements identification.**

A *H. parviporum-*specific *de novo* repetitive sequences library was constructed using RepeatScout v.1.0.5 (-l 14) [1]. The consensus sequences in the library were filtered by following procedures: 1). Low-complexity and tandem repeats were removed using Nseg [2] and TRF [3]; 2). Repeats occurring less than 10 copies in the genome were eliminated; 3). Repeats less than 100 bp were filtered out. The filtered library thereafter was blastx against Swiss-Prot manually annotated protein database v.21.9.2016. Sequence fragments having significant hits except for TE-related proteins were excluded along with 50 bp flanking sequence of the fragments. The remaining sequences less than 50 bp were also removed. The resulting consensus sequences were classified by tblastx against Repbase Update database v.29.8.2016 and REPCLASS v.1.0.1 pipeline [4], which automates the classification of TEs based on homology, target-site duplication, and structure features such as long terminal repeats and terminal inverted repeats.

Full length of recent (above 99% similarity in two terminal repeats) and relatively old long- terminal-repeat (LTR) retrotransposons (85%-99% similarity in two terminal repeats) were predicted and filtered by LTRharvest [5] and LTRdigest [6] in GenomicTools v.1.5.9 [7]. Putative LTR retrotransposons were filtered if 1). Nested insertions of other LTR sequences were found; 2). 50 % of polypurine tract (PPT) or primer binding site (PBS) were not present in the internal region of the elements and the distance between terminal repeats and PBS or PPT was larger than 20 bp; 3). 50 bp flanking sequence of 5’LTR and 3’LTR were alignable (60% identity as the threshold). The filtered LTR elements were then classified by tblastx against Repbase Update database. The consensus sequences from RepeatScout pipeline and full length classified LTR elements were combined and formatted as the input for RepeatMasker v.4.0.6 (<http://www.repeatmasker.org/>) for soft-masking. The statistics of masked TEs in the assembly were obtained through a perl script from Parsing-RepeatMasker-Outputs (<https://github.com/4ureliek/Parsing-RepeatMasker-Outputs>).

**References**

1. Price AL, Jones NC, Pevzner PA: **De novo identification of repeat families in large genomes**. *Bioinformatics* 2005, **21**:I351-I358.

2. Wootton JC, Federhen S: **Analysis of compositionally biased regions in sequence databases**. *Method Enzymol* 1996, **266**:554-571.

3. Benson G: **Tandem repeats finder: a program to analyze DNA sequences**. *Nucleic Acids Res* 1999, **27**(2):573-580.

4. Feschotte C, Keswani U, Ranganathan N, Guibotsy ML, Levine D: **Exploring Repetitive DNA Landscapes Using REPCLASS, a Tool That Automates the Classification of Transposable Elements in Eukaryotic Genomes**. *Genome Biol Evol* 2009, **1**:205-220.

5. Ellinghaus D, Kurtz S, Willhoeft U: **LTRharvest, an efficient and flexible software for de novo detection of LTR retrotransposons**. *Bmc Bioinformatics* 2008, **9**:18.

6. Steinbiss S, Willhoeft U, Gremme G, Kurtz S: **Fine-grained annotation and classification of de novo predicted LTR retrotransposons**. *Nucleic Acids Res* 2009, **37**(21):7002-7013.

7. Gremme G, Steinbiss S, Kurtz S: **GenomeTools: A Comprehensive Software Library for Efficient Processing of Structured Genome Annotations**. *Ieee Acm T Comput Bi* 2013, **10**(3):645-656.
