# Supplementary figures and images for: Intraspecific comparative genomics of isolates of the Norway spruce pathogen (Heterobasidion parviporum) and identification of its potential virulence factors
Source: BMC Genomics. 2018 Mar 27;19:220. doi: 10.1186/s12864-018-4610-4 (PMC5870257; doi:10.1186/s12864-018-4610-4)

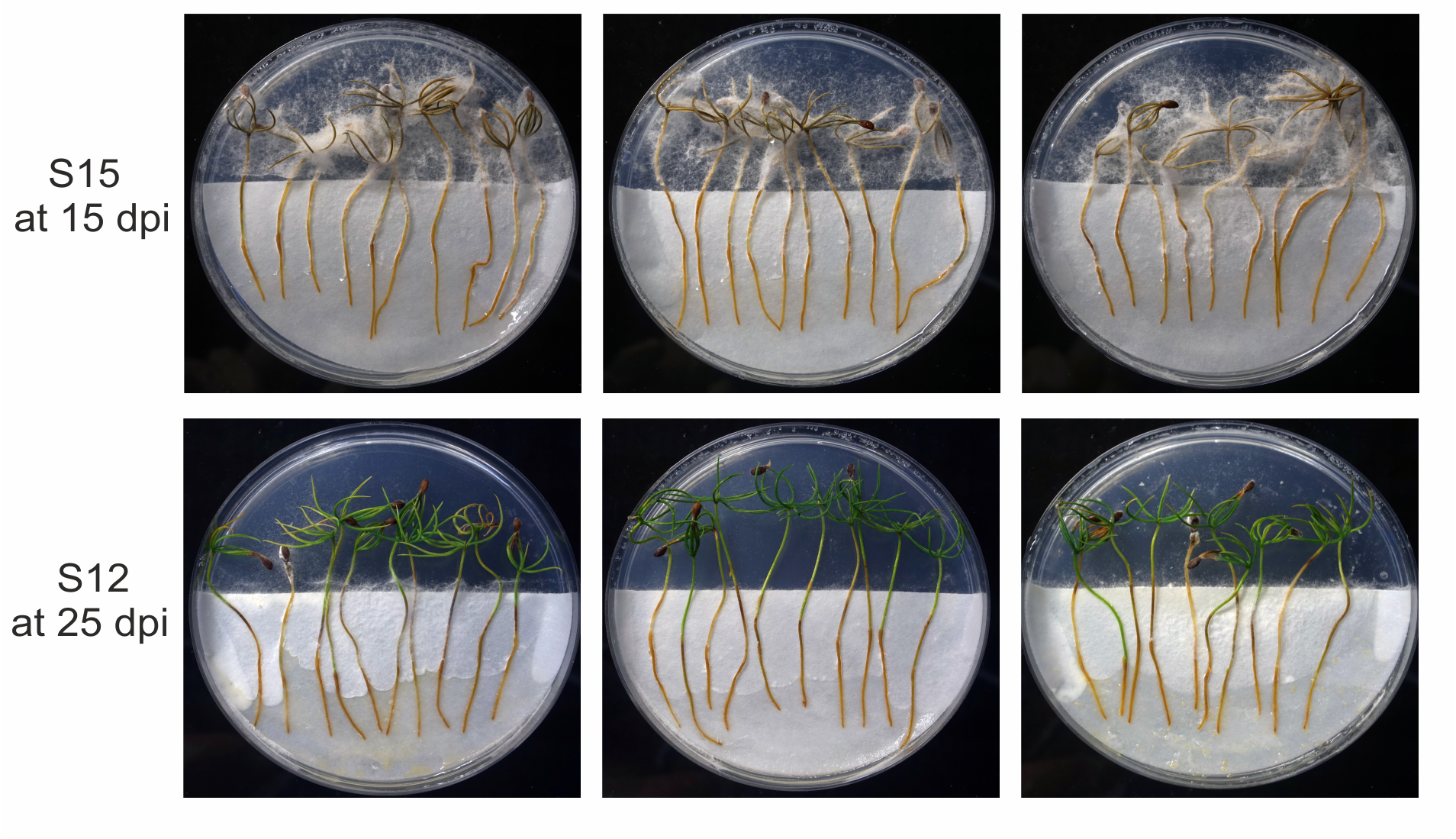

Supplement: Supplementary file 3 — Figure S1. Norway spruce seedlings infected by isolate S15 (upper row) and S12 (lower row) at 15 dpi and 25 dpi respectively in virulence assay. (TIFF 2918 kb) [file 12864_2018_4610_MOESM3_ESM.tif]

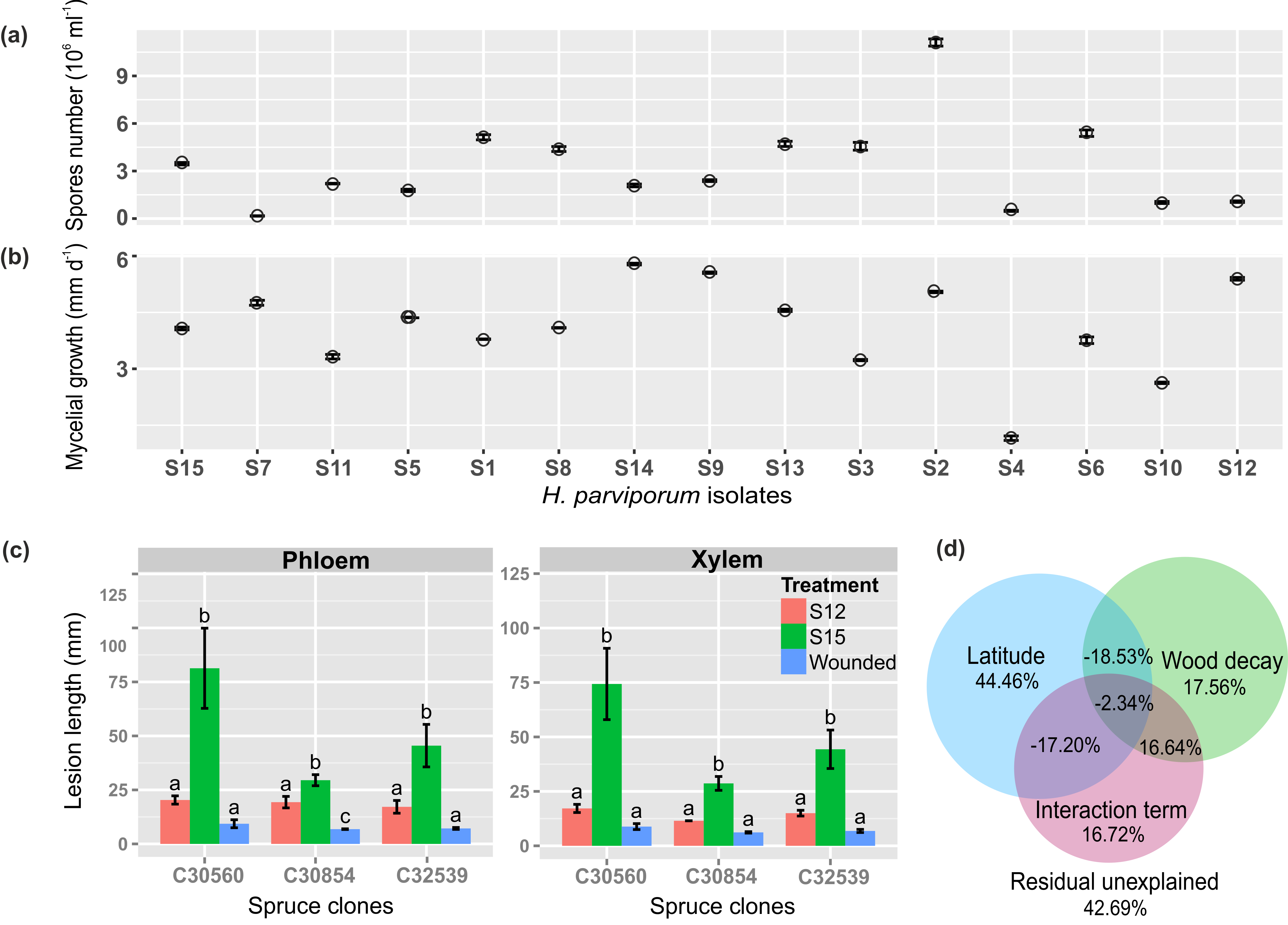

Supplement: Supplementary file 4 — Figure S2. (a) Sporulation and (b) Vegetative growth rate of 15 H. parviporum isolates. Isolates were sorted by mortality rate at 25 dpi in descending order. (c) Lesion lengths in phloem and xylem caused by isolate S12, isolate S15 and wounding treatments in virulence validation. Different lowercase letters indicate statistically significant differences (P < 0.001). Error bars stand for standard errors. (d) Variation partitioned by wood decay, latitude and their interaction term. (TIFF 1303 kb) [file 12864_2018_4610_MOESM4_ESM.tif]

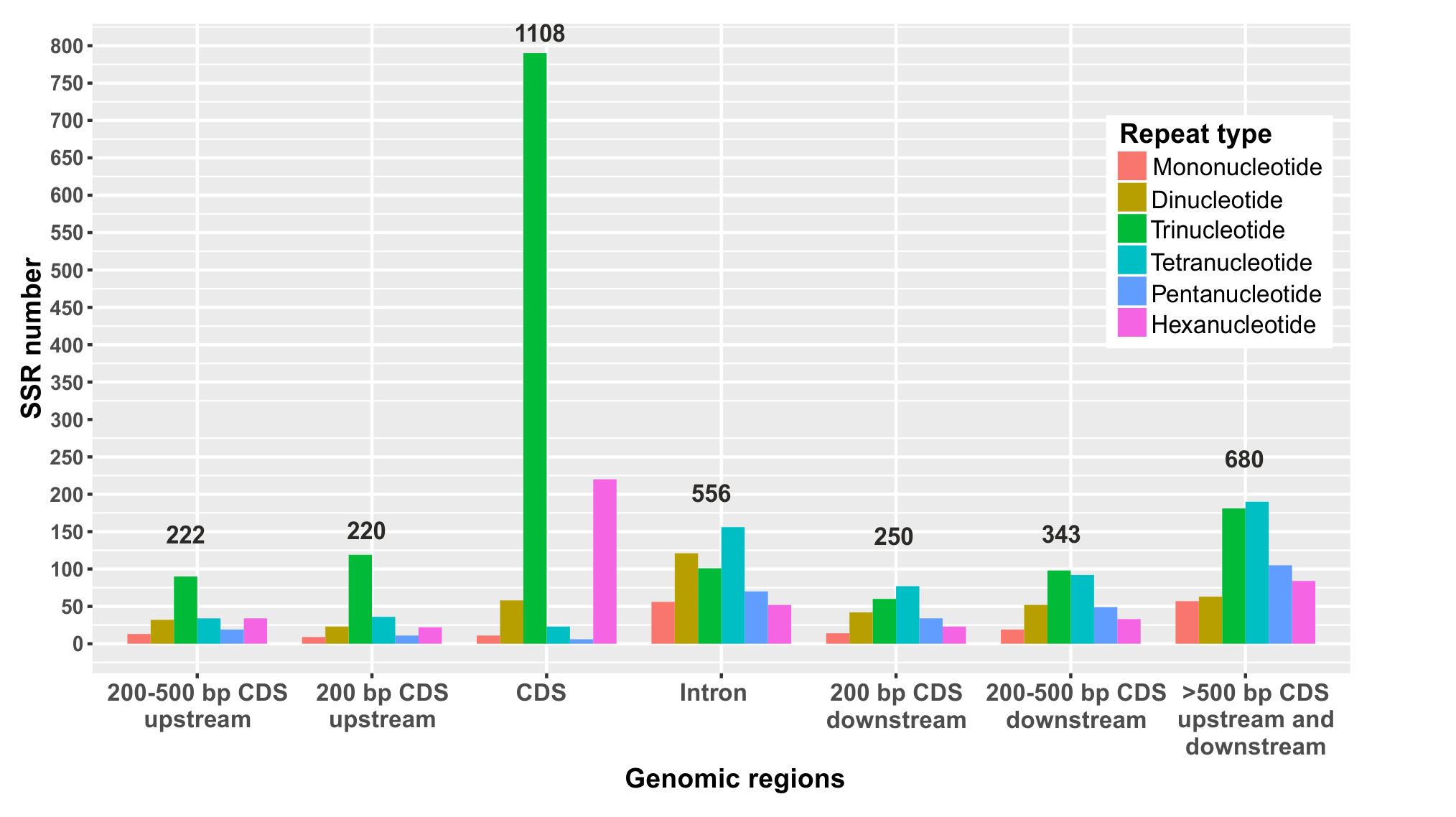

Supplement: Supplementary file 8 — Figure S3 Simple sequence repeats (SSRs) distribution in selected genomic regions of S15. Numbers on the bars represent the total SSRs number in that particular type of region. (TIFF 321 kb) [file 12864_2018_4610_MOESM8_ESM.tif]

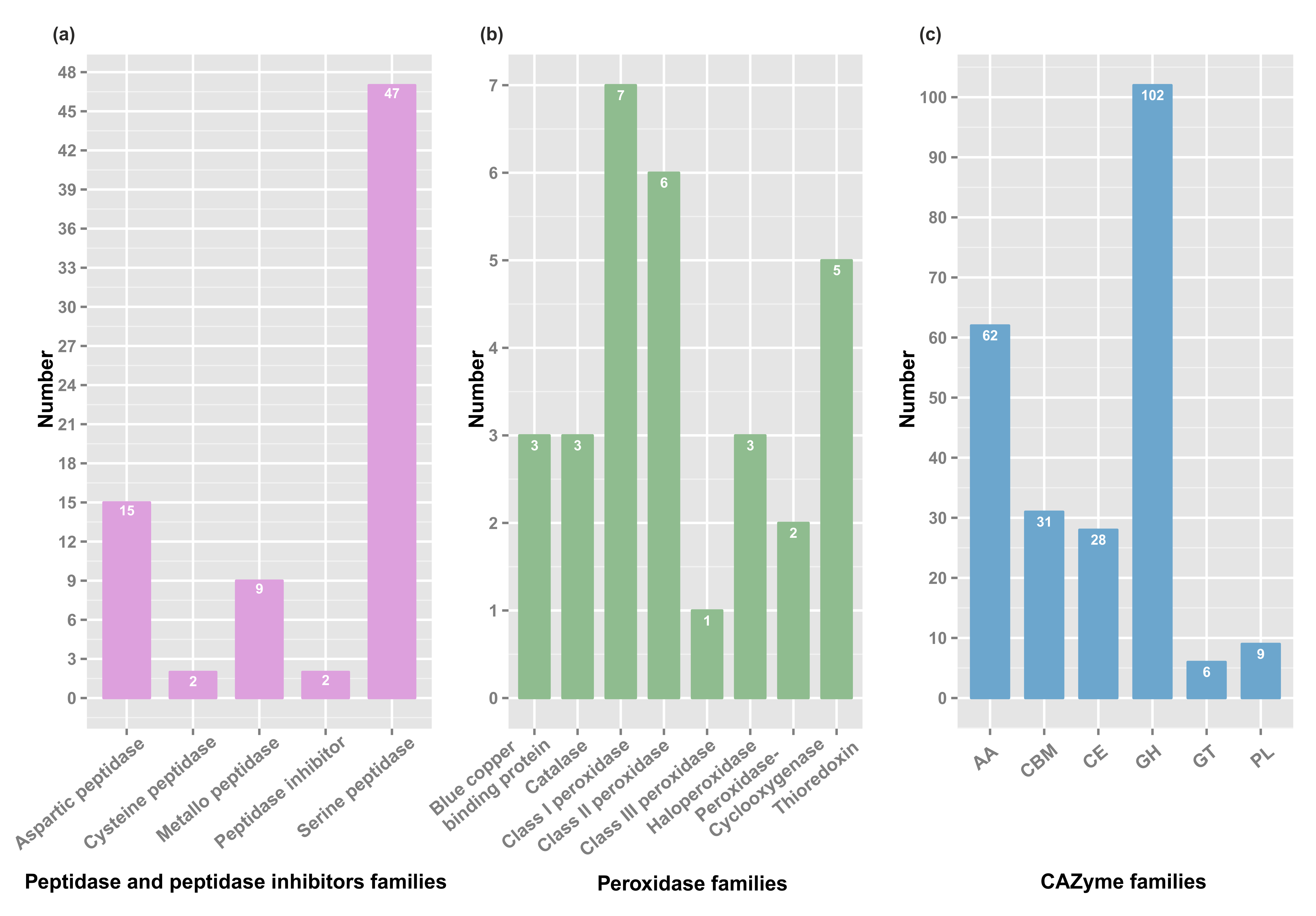

Supplement: Supplementary file 10 — Figure S4 Identified families of peptidase and peptidase inhibitors, peroxidases and CAZymes in isolate S15 secretome. (TIFF 1667 kb) [file 12864_2018_4610_MOESM10_ESM.tif]

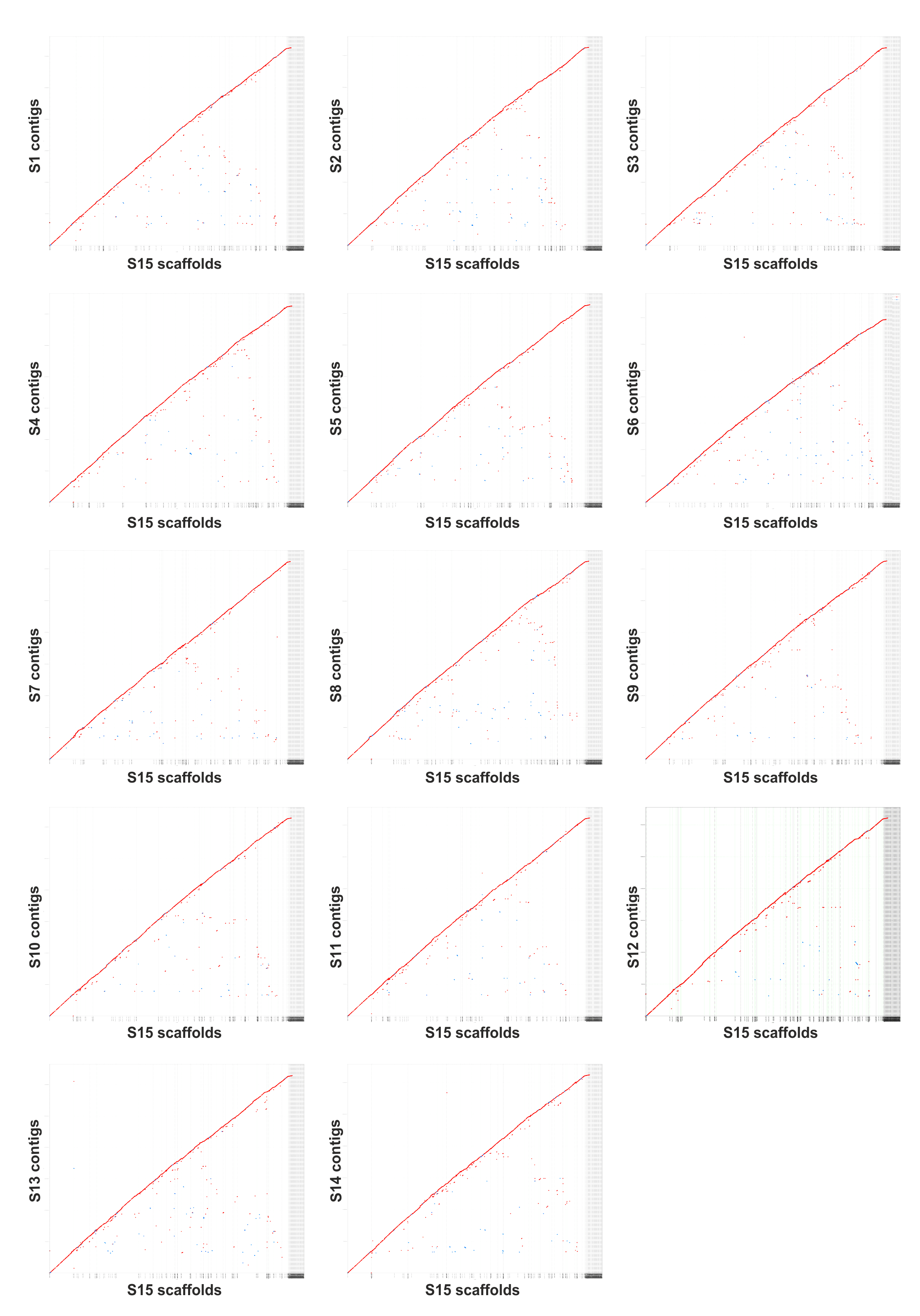

Supplement: Supplementary file 14 — Figure S5. Genome alignment between H. parviporum isolate S15 scaffolds and the contigs of re-sequenced isolates. The dot plot represents one to one best mapping, and dots on the diagonal denote co-linearity between the two genomes. Red dots stand for matches in the forward direction and blue dots are inversions relative to S15 scaffolds. (TIFF 2132 kb) [file 12864_2018_4610_MOESM14_ESM.tif]

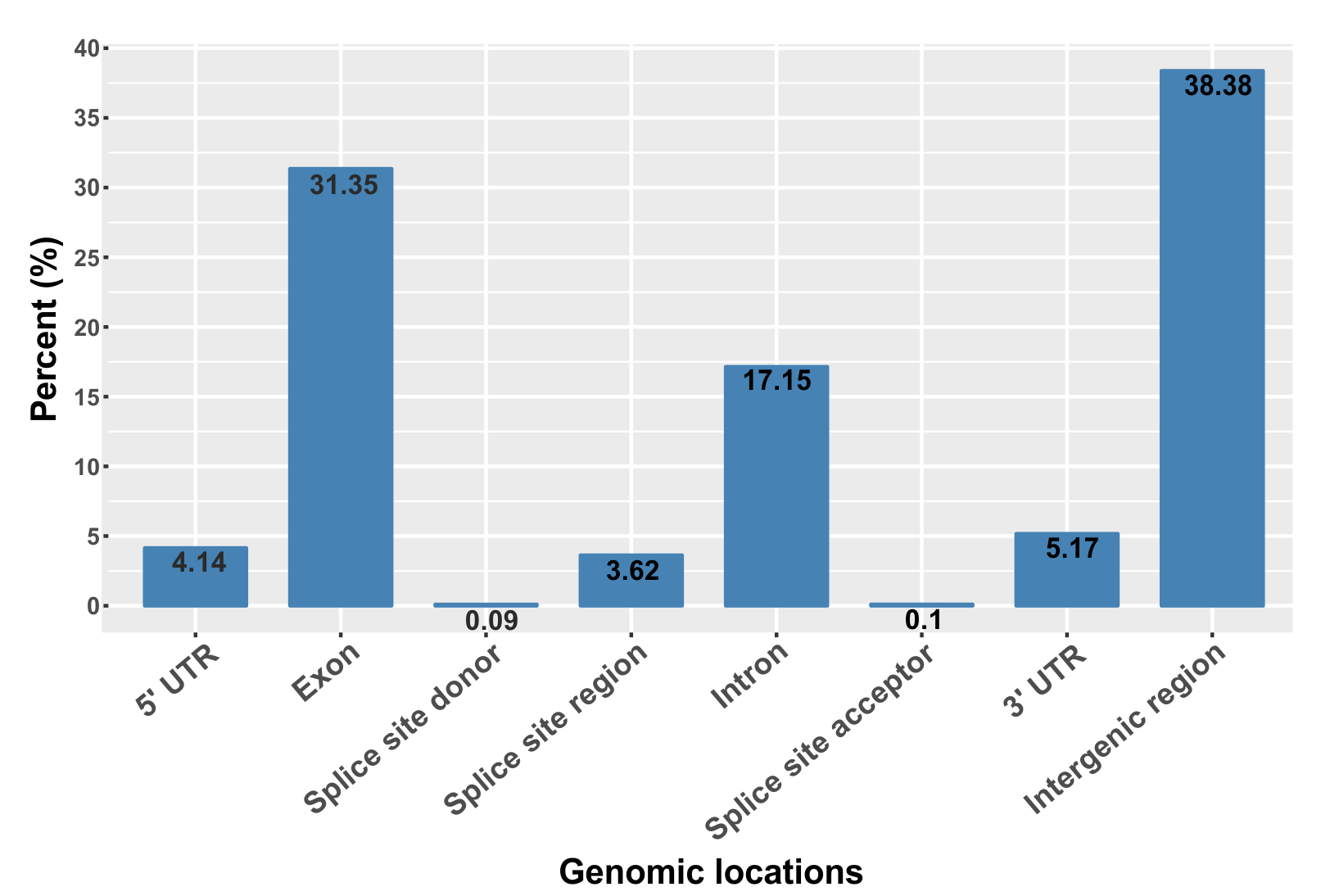

Supplement: Supplementary file 15 — Figure S6. Variant distributions in different type of genomic regions. (TIFF 211 kb) [file 12864_2018_4610_MOESM15_ESM.tif]

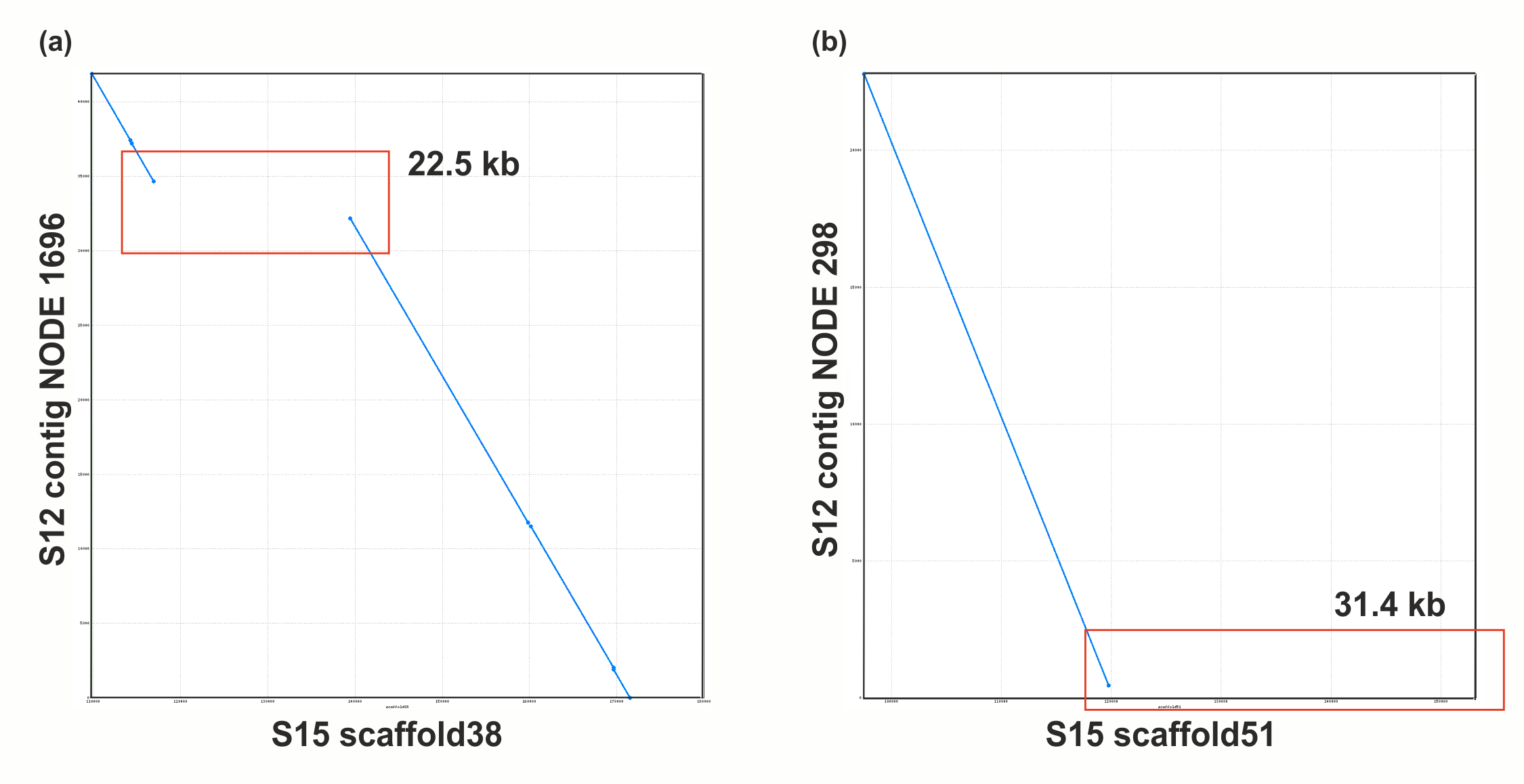

Supplement: Supplementary file 19 — Figure S7. Dot plots of alignments of S12 contigs to the corresponding regions of reference (a) scaffold38 and (b) scaffold51. (TIFF 301 kb) [file 12864_2018_4610_MOESM19_ESM.tif]
