# Supplementary material for: Intraspecific comparative genomics of isolates of the Norway spruce pathogen (Heterobasidion parviporum) and identification of its potential virulence factors
Source: BMC Genomics. 2018 Mar 27;19:220. doi: 10.1186/s12864-018-4610-4 (PMC5870257; doi:10.1186/s12864-018-4610-4)
Supplement: Supplementary file 5 — Table S1. Summary of transposable elements in S15. (DOCX 14 kb) [file 12864_2018_4610_MOESM5_ESM.docx]

**Table S1. Summary of transposable elements in S15.**

| **Classes** | **Families** | **Number of**  **consensus sequences** | **Number of occurrences** | **Length occupied1 (bp)** | **Assembly coverage (%)** |
| --- | --- | --- | --- | --- | --- |
| Non-LTR  retrotransposons | Tad1 | 2 | 24 | 36,254 | 0.10 |
|  | Crack | 1 | 15 | 16,946 | 0.04 |
|  | Other | 4 | 97 | 52,799 | 0.14 |
| LTR retrotransposons | Gypsy | 41 | 1665 | 5,042,790 | 13.36 |
|  | Copia | 21 | 474 | 388,510 | 1.03 |
|  | DIRs | 2 | 46 | 111,100 | 0.29 |
|  | Other | 1 | 9 | 2,205 | 0.01 |
| DNA transposons | Helitron | 5 | 214 | 104,363 | 0.28 |
|  | Harbinger | 1 | 57 | 25,517 | 0.07 |
|  | Mariner | 1 | 20 | 21,580 | 0.06 |
|  | Maverick | 1 | 19 | 10,859 | 0.03 |
|  | Merlin | 2 | 80 | 40,422 | 0.11 |
|  | Other | 13 | 329 | 144,193 | 0.38 |
| Uncategorized | - | 172 | 3537 | 1,896,254 | 5.02 |
| Total | - | 267 | 6586 | 7,893,792 (7,660,713)2 | 20.91 (20.29)2 |

1Overlapping length between repetitive elements from the same family was subtracted.

2Numbers in parentheses are the ultimate values after overlapping elements from the same and different families were subtracted.
