# Supplementary material for: Intraspecific comparative genomics of isolates of the Norway spruce pathogen (Heterobasidion parviporum) and identification of its potential virulence factors
Source: BMC Genomics. 2018 Mar 27;19:220. doi: 10.1186/s12864-018-4610-4 (PMC5870257; doi:10.1186/s12864-018-4610-4)
Supplement: Supplementary file 6 — Table S2. Summary of simple sequence repeats (SSRs) in S15. (DOCX 12 kb) [file 12864_2018_4610_MOESM6_ESM.docx]

**Table S2. Summary of simple sequence repeats in S15.**

| **Repeat type** | **Counts** | **Average length (bp)** | **Counts/Mb** |
| --- | --- | --- | --- |
| Mononucleotide | 160 | 16.59 | 4.24 |
| Dinucleotide | 351 | 16.89 | 9.30 |
| Trinucleotide | 1295 | 19.76 | 34.30 |
| Tetranucleotide | 569 | 20.53 | 15.07 |
| Pentanucleotide | 267 | 42.84 | 7.07 |
| Hexanucleotide | 424 | 30.48 | 11.23 |
