# Supplementary material for: Intraspecific comparative genomics of isolates of the Norway spruce pathogen (Heterobasidion parviporum) and identification of its potential virulence factors
Source: BMC Genomics. 2018 Mar 27;19:220. doi: 10.1186/s12864-018-4610-4 (PMC5870257; doi:10.1186/s12864-018-4610-4)
Supplement: Supplementary file 7 — Table S3. The most frequent fully standardized SSR motifs in S15. (DOCX 12 kb) [file 12864_2018_4610_MOESM7_ESM.docx]

**Table S3. The most frequent fully standardized SSR motifs in S15.**

| **Motif** | **Counts** | **Average length (bp)** | **Counts/Mb** |
| --- | --- | --- | --- |
| CCG | 298 | 18.14 | 7.89 |
| AGC | 269 | 19.91 | 7.12 |
| ACG | 262 | 19.25 | 6.94 |
| AGG | 154 | 20.86 | 4.08 |
| AG | 131 | 17.99 | 3.47 |
| ACC | 126 | 20.91 | 3.34 |
| CG | 122 | 15.09 | 3.23 |
| C | 109 | 16.86 | 2.89 |
