# Supplementary material for: Intraspecific comparative genomics of isolates of the Norway spruce pathogen (Heterobasidion parviporum) and identification of its potential virulence factors
Source: BMC Genomics. 2018 Mar 27;19:220. doi: 10.1186/s12864-018-4610-4 (PMC5870257; doi:10.1186/s12864-018-4610-4)
Supplement: Supplementary file 9 — Table S4. Summary of CAZymes in S15 proteome. (DOCX 17 kb) [file 12864_2018_4610_MOESM9_ESM.docx]

**Table S4. Summary of CAZymes in S15 proteome.**

| **CAZyme**  **families1** | **Number** | **CAZyme**  **families** | **Number** | **CAZyme**  **families** | **Number** | **CAZyme**  **families** | **Number** |
| --- | --- | --- | --- | --- | --- | --- | --- |
| GH families |  | GH72 | 1 | CBM families |  | GT33 | 1 |
| GH2 | 3 | GH74 | 2 | CBM1 | 17 | GT35 | 1 |
| GH3 | 12 | GH76 | 2 | CBM5 | 4 | GT39 | 3 |
| GH5 | 16 | GH78 | 2 | CBM12 | 1 | GT48 | 2 |
| GH6 | 1 | GH79 | 9 | CBM13 | 4 | GT49 | 1 |
| GH7 | 1 | GH81 | 1 | CBM18 | 2 | GT50 | 1 |
| GH9 | 1 | GH85 | 1 | CBM19 | 4 | GT57 | 3 |
| GH10 | 2 | GH88 | 1 | CBM20 | 3 | GT58 | 1 |
| GH12 | 3 | GH92 | 3 | CBM21 | 2 | GT59 | 1 |
| GH13 | 8 | GH95 | 1 | CBM32 | 1 | GT65 | 1 |
| GH15 | 4 | GH99 | 1 | CBM35 | 2 | GT66 | 1 |
| GH16 | 28 | GH105 | 4 | CBM41 | 1 | GT68 | 2 |
| GH17 | 2 | GH109 | 7 | CBM43 | 1 | GT69 | 3 |
| GH18 | 11 | GH115 | 1 | CBM48 | 1 | GT76 | 1 |
| GH20 | 4 | GH125 | 1 | CBM50 | 8 | GT90 | 1 |
| GH23 | 1 | GH127 | 1 | GT families |  | PL families |  |
| GH27 | 4 | GH128 | 1 | GT1 | 2 | PL1 | 3 |
| GH28 | 8 | GH131 | 2 | GT2 | 13 | PL3 | 1 |
| GH29 | 2 | GH133 | 1 | GT3 | 1 | PL4 | 1 |
| GH30 | 2 | CE families |  | GT4 | 3 | PL8 | 2 |
| GH31 | 4 | CE1 | 14 | GT5 | 1 | PL14 | 4 |
| GH32 | 1 | CE2 | 1 | GT8 | 5 | AA families |  |
| GH35 | 4 | CE4 | 7 | GT15 | 4 | AA1 | 13 |
| GH37 | 2 | CE5 | 1 | GT17 | 1 | AA2 | 12 |
| GH38 | 1 | CE8 | 3 | GT20 | 3 | AA3 | 41 |
| GH43 | 7 | CE9 | 2 | GT21 | 1 | AA4 | 2 |
| GH45 | 1 | CE10 | 48 | GT22 | 3 | AA5 | 5 |
| GH47 | 6 | CE12 | 3 | GT23 | 1 | AA6 | 2 |
| GH51 | 2 | CE14 | 1 | GT24 | 1 | AA7 | 22 |
| GH53 | 1 | CE15 | 1 | GT25 | 1 | AA8 | 2 |
| GH55 | 1 | CE16 | 8 | GT28 | 1 | AA9 | 10 |
| GH63 | 2 |  |  | GT31 | 1 | AA11 | 2 |
| GH71 | 2 |  |  | GT32 | 3 |  |  |

1GH: Glycoside hydrolases, CE: Carbohydrate esterases, CBM: Carbohydrate-binding module, PL: Polysaccharide lyases, GT: Glycosyltransferases, AA: Auxiliary activities.
