# Supplementary material for: Intraspecific comparative genomics of isolates of the Norway spruce pathogen (Heterobasidion parviporum) and identification of its potential virulence factors
Source: BMC Genomics. 2018 Mar 27;19:220. doi: 10.1186/s12864-018-4610-4 (PMC5870257; doi:10.1186/s12864-018-4610-4)
Supplement: Supplementary file 11 — Table S5. Summary of peptidases, peptidase inhibitors and peroxidases in S15 secretome. (DOCX 14 kb) [file 12864_2018_4610_MOESM11_ESM.docx]

**Table S5. Summary of peptidases, peptidase inhibitors and peroxidases in S15 secretome**

| **Peptidase families1** | **Number2** | **Peroxidase (super)families3** | **Number2** |
| --- | --- | --- | --- |
| Aspartic families |  | Thioredoxin families |  |
| A1A | 15 (15) | Thioredoxin type M (TrxM) | 3 (3) |
| Cysteine families |  | Thioredoxin type O (TrxO) | 1 |
| C13 | 1 | Thioredoxin type Clot (Clot) | 1 (1) |
| C14B | 1 | Blue copper binding protein families |  |
| Metallo families |  | Early nodulin (ENODL) | 3 (2) |
| M14A | 1 | Haloperoxidase superfamilies |  |
| M28A | 3 | No haem, no metal haloperoxidase (HalNPrx) | 2 |
| M28E | 1 | Haloperoxidase (HalPrx) | 1 (1) |
| M35 | 1 | Catalase family |  |
| M36 | 1 (1) | Catalase (Kat) | 3 (1) |
| M43B | 1 (1) | Class I peroxidase superfamilies |  |
| M67X | 1 | Cytochrome C peroxidase (CcP) | 2 |
| Serine family |  | Ascorbate peroxidase (APx) | 5 (5) |
| S1E | 1 | Class II peroxidase families |  |
| S8A | 4 (3) | Manganese peroxidase | 5 (4) |
| S9X | 13 | Other class II peroxidase type B | 1 (1) |
| S9C | 1 | Class III peroxidase families |  |
| S10 | 9 | Class III peroxidase (Prx) | 1 |
| S12 | 2 | Peroxidase-Cyclooxygenase superfamily |  |
| S28 | 3 | Linoleate diol synthase (LDS) | 2 (2) |
| S33 | 2 |  |  |
| S53 | 12 |  |  |
| **Peptidase Inhibitors** |  |  |  |
| I51 | 2 |  |  |

1Classification based on MEROPS database.

2Number in parentheses are the number of proteins having PHI-base hits with annotations of “reduced virulence”, “loss of pathogenicity” or “effector_(plant_avirulence_determinant).

3Classification based on PeroxiBase database
