# Supplementary material for: Intraspecific comparative genomics of isolates of the Norway spruce pathogen (Heterobasidion parviporum) and identification of its potential virulence factors
Source: BMC Genomics. 2018 Mar 27;19:220. doi: 10.1186/s12864-018-4610-4 (PMC5870257; doi:10.1186/s12864-018-4610-4)
Supplement: Supplementary file 12 — Table S6. Summary of CAZymes in S15 secretome. (DOCX 15 kb) [file 12864_2018_4610_MOESM12_ESM.docx]

**Table S6. Summary of CAZymes in S15 secretome.**

| **CAZyme**  **families1** | **Number2** | **CAZyme**  **families** | **Number** | **CAZyme**  **families** | **Number** | **CAZyme**  **families** | **Number** |
| --- | --- | --- | --- | --- | --- | --- | --- |
| GH families |  | GH37 | 1 (1) | CE families |  | PL families |  |
| GH2 | 1 | GH43 | 4 | CE1 | 1 (1) | PL1 | 3 (3) |
| GH3 | 7 | GH45 | 1 | CE2 | 1 | PL4 | 1 |
| GH5 | 8 (2) | GH47 | 3 | CE4 | 3 (3) | PL8 | 2 (1) |
| GH6 | 1 (1) | GH51 | 2 | CE5 | 1 (1) | PL14 | 3 |
| GH10 | 2 (2) | GH53 | 1 | CE8 | 2 | GT families |  |
| GH12 | 2 (2) | GH55 | 1 | CE10 | 14 | GT2 | 1 (1) |
| GH13 | 3 (1) | GH63 | 1 | CE12 | 1 | GT15 | 3 (3) |
| GH15 | 1 | GH72 | 1 (1) | CE15 | 1 | GT24 | 1 (1) |
| GH16 | 15 | GH74 | 1 (1) | CE16 | 4 | GT25 | 1 |
| GH17 | 1 | GH76 | 1 | CBM families |  | AA families |  |
| GH18 | 10 (6) | GH78 | 1 | CBM1 | 13 (9) | AA1 | 12 (12) |
| GH20 | 3 (3) | GH79 | 5 | CBM5 | 4 (2) | AA2 | 8 (7) |
| GH27 | 1 | GH81 | 1 | CBM18 | 2 (1) | AA3 | 20 (20) |
| GH28 | 5 (4) | GH88 | 1 | CBM19 | 4 | AA5 | 3 (3) |
| GH30 | 2 | GH92 | 3 | CBM20 | 1 | AA7 | 7 (7) |
| GH31 | 4 (4) | GH105 | 3 | CBM35 | 2 | AA8 | 1 (1) |
| GH32 | 1 | GH127 | 1 | CBM43 | 1 (1) | AA9 | 10 (10) |
| GH35 | 2 | GH128 | 1 | CBM50 | 4 | AA11 | 1 |

1GH: Glycoside hydrolases, CE: Carbohydrate esterases, CBM: Carbohydrate-binding module, PL: Polysaccharide lyases, GT: Glycosyltransferases, AA: Auxiliary activities.

2Number in parentheses are the number of proteins having PHI-base hits with annotations of “reduced virulence”, “loss of pathogenicity” or “effector_(plant_avirulence_determinant).
