# Supplementary material for: Intraspecific comparative genomics of isolates of the Norway spruce pathogen (Heterobasidion parviporum) and identification of its potential virulence factors
Source: BMC Genomics. 2018 Mar 27;19:220. doi: 10.1186/s12864-018-4610-4 (PMC5870257; doi:10.1186/s12864-018-4610-4)
Supplement: Supplementary file 13 — Table S7. Repertoire of CAZymes targeting plant cell walls (PCW) and lignin in S15 secretome. (DOCX 14 kb) [file 12864_2018_4610_MOESM13_ESM.docx]

**Table S7. Repertoire of CAZymes targeting plant cell walls (PCW) and lignin in S15 secretome**.

| **CAZyme**  **families** | **Substrates** | **Number1** | **CAZyme**  **families** | **Substrates** | **Number** |
| --- | --- | --- | --- | --- | --- |
| GH6 | PCW-Cellulose | 1 (1) | GH43 | PCW-Hemicellulose-Pectin | 4 |
| GH12 | PCW-Cellulose | 2 (2) | GH51 | PCW-Hemicellulose-Pectin | 2 |
| GH45 | PCW-Cellulose | 1 | GH53 | PCW-Hemicellulose-Pectin | 1 |
| GH74 | PCW-Cellulose | 1 (1) | CE12 | PCW-Hemicellulose-Pectin | 1 |
| AA9 | PCW-Cellulose | 10 (10) |  | Subtotal | 8 |
|  | Subtotal | 15 (14) | GH28 | PCW-Pectin | 5 (4) |
| GH10 | PCW-Hemicellulose | 2 (2) | GH78 | PCW-Pectin | 1 |
| GH27 | PCW-Hemicellulose | 1 | GH88 | PCW-Pectin | 1 |
| GH31 | PCW-Hemicellulose | 4 (4) | GH105 | PCW-Pectin | 3 |
| GH35 | PCW-Hemicellulose | 2 | PL1 | PCW-Pectin | 3 (3) |
| CE1 | PCW-Hemicellulose | 1 (1) | PL4 | PCW-Pectin | 1 |
| CE2 | PCW-Hemicellulose | 1 | CE8 | PCW-Pectin | 2 |
| CE5 | PCW-Hemicellulose | 1 (1) |  | Subtotal | 16 (7) |
| CE15 | PCW-Hemicellulose | 1 | AA13 | Lignin | 12 (12) |
| CE16 | PCW-Hemicellulose | 4 | AA24 | Lignin | 8 (7) |
|  | Subtotal | 17 (8) | AA35 | Lignin | 20 (20) |
| GH3 | Cell wall in general2 | 7 | AA56 | Lignin | 3 (3) |
| GH5 | Cell wall in general | 8 (2) |  | Subtotal | 43 (42) |

1Number in parentheses are the number of proteins having PHI-base hits with annotations of “reduced virulence”, “loss of pathogenicity” or “effector_(plant_avirulence_determinant).

2Enzymes that can act both on plant and fungal cell walls.

3Multicopper oxidases (including laccases).

4Manganese peroxidases and other heme-containing peroxidases.

5Glucose-methanol-choline (GMC) oxidoreductases (including aryl alcohol oxidases, choline dehydrogenase).

6Copper radical oxidases (including glyoxal oxidases).
