# Supplementary material for: Intraspecific comparative genomics of isolates of the Norway spruce pathogen (Heterobasidion parviporum) and identification of its potential virulence factors
Source: BMC Genomics. 2018 Mar 27;19:220. doi: 10.1186/s12864-018-4610-4 (PMC5870257; doi:10.1186/s12864-018-4610-4)
Supplement: Supplementary file 16 — Table S8. Nucleotides counts and their frequencies within 1 bp flanking C-to-T mutations. (DOCX 13 kb) [file 12864_2018_4610_MOESM16_ESM.docx]

**Table S8. Nucleotides counts and their frequencies within 1 bp flanking C-to-T mutations.**

|  | **Counts (freq)2** |  | **Counts (freq)** |  | **Counts (freq)** |  | **Counts (freq)** |
| --- | --- | --- | --- | --- | --- | --- | --- |
| ACA | 6990 (0.060) | ACC | 4860 (0.042) | ACG | 11684 (0.100) | ACT | 3473 (0.030) |
| CCA | 7659 (0.066) | CCC | 6130 (0.053) | CCG | 10144 (0.087) | CCT | 5575 (0.048) |
| GCA | 5527 (0.047) | GCC | 5198 (0.045) | GCG | 12100 (0.104) | GCT | 4350 (0.037) |
| TCA | 7639 (0.066) | TCC | 6338 (0.054) | TCG | 13070 (0.112) | TCT | 5710 (0.049) |
| nCA1 | 27815 (0.239) | nCC | 22526 (0.193) | nCG | 46998 (0.404) | nCT | 19108 (0.164) |

1n indicates A, C, G and T;

2freq denotes frequency out of all C-to-T mutations.
