# Supplementary material for: Intraspecific comparative genomics of isolates of the Norway spruce pathogen (Heterobasidion parviporum) and identification of its potential virulence factors
Source: BMC Genomics. 2018 Mar 27;19:220. doi: 10.1186/s12864-018-4610-4 (PMC5870257; doi:10.1186/s12864-018-4610-4)
Supplement: Supplementary file 20 — Table S11. Significantly over-represented GO terms of conserved core genes compared with all genes in S15. (DOCX 19 kb) [file 12864_2018_4610_MOESM20_ESM.docx]

**Table S11 Significantly over-represented GO terms of conserved core genes against all genes in S15**

| **GO IDs** | **GO annotation** | **GO**  **type1** | **FDR2** | **P-value** | **Genes in test**  **set** | **Genes in reference**  **set** |
| --- | --- | --- | --- | --- | --- | --- |
| GO:0000122 | negative regulation of transcription from RNA polymerase II promoter | BP | 0.0064 | 1.55E-04 | 11 | 13 |
| GO:0000398 | mRNA splicing, via spliceosome | BP | 0.0176 | 5.47E-04 | 17 | 36 |
| GO:0000722 | telomere maintenance via recombination | BP | 0.0388 | 0.0016 | 4 | 1 |
| GO:0006342 | chromatin silencing | BP | 0.0267 | 9.42E-04 | 15 | 31 |
| GO:0006417 | regulation of translation | BP | 0.0407 | 0.0018 | 24 | 70 |
| GO:0006511 | ubiquitin-dependent protein catabolic process | BP | 0.0313 | 0.0012 | 23 | 63 |
| GO:0006633 | fatty acid biosynthetic process | BP | 1.22E-10 | 7.27E-13 | 29 | 22 |
| GO:0006730 | one-carbon metabolic process | BP | 1.60E-04 | 2.51E-06 | 15 | 15 |
| GO:0007096 | regulation of exit from mitosis | BP | 0.0392 | 0.0017 | 6 | 5 |
| GO:0008298 | intracellular mRNA localization | BP | 0.0388 | 0.0016 | 4 | 1 |
| GO:0009226 | nucleotide-sugar biosynthetic process | BP | 0.0247 | 8.40E-04 | 5 | 2 |
| GO:0015940 | pantothenate biosynthetic process | BP | 9.15E-12 | 3.74E-14 | 29 | 18 |
| GO:0019220 | regulation of phosphate metabolic process | BP | 0.0402 | 0.0018 | 14 | 30 |
| GO:0022618 | ribonucleoprotein complex assembly | BP | 0.0095 | 2.48E-04 | 19 | 40 |
| GO:0031497 | chromatin assembly | BP | 0.0173 | 5.22E-04 | 10 | 13 |
| GO:0032506 | cytokinetic process | BP | 0.0391 | 0.0017 | 9 | 13 |
| GO:0033365 | protein localization to organelle | BP | 0.0095 | 2.51E-04 | 31 | 87 |
| GO:0034243 | regulation of transcription elongation from RNA polymerase II promoter | BP | 0.0027 | 5.70E-05 | 11 | 11 |
| GO:0034314 | Arp2/3 complex-mediated actin nucleation | BP | 0.0257 | 8.98E-04 | 6 | 4 |
| GO:0042254 | ribosome biogenesis | BP | 1.29E-08 | 1.02E-10 | 75 | 183 |
| GO:0042797 | tRNA transcription from RNA polymerase III promoter | BP | 0.0392 | 0.0017 | 6 | 5 |
| GO:0042967 | acyl-carrier-protein biosynthetic process | BP | 2.29E-09 | 1.62E-11 | 38 | 50 |
| GO:0045944 | positive regulation of transcription from RNA polymerase II promoter | BP | 5.39E-04 | 9.32E-06 | 19 | 29 |
| GO:0051123 | RNA polymerase II transcriptional preinitiation complex assembly | BP | 0.0392 | 0.0017 | 6 | 5 |
| GO:0051338 | regulation of transferase activity | BP | 0.0478 | 0.0023 | 10 | 17 |
| GO:0051574 | positive regulation of histone H3-K9 methylation | BP | 0.0424 | 0.0019 | 5 | 3 |
| GO:0060260 | regulation of transcription initiation from RNA polymerase II promoter | BP | 0.0354 | 0.0014 | 7 | 7 |
| GO:0065002 | intracellular protein transmembrane transport | BP | 0.0229 | 7.45E-04 | 12 | 20 |
| GO:0070682 | proteasome regulatory particle assembly | BP | 0.0424 | 0.0020 | 5 | 3 |
| GO:1902412 | regulation of mitotic cytokinesis | BP | 0.0257 | 8.98E-04 | 6 | 4 |
| GO:1905268 | negative regulation of chromatin organization | BP | 0.0234 | 7.77E-04 | 7 | 6 |
| GO:1990542 | mitochondrial transmembrane transport | BP | 0.0391 | 0.0017 | 9 | 13 |
| GO:0005666 | DNA-directed RNA polymerase III complex | CC | 0.0392 | 0.0017 | 6 | 5 |
| GO:0005761 | mitochondrial ribosome | CC | 0.0391 | 0.0017 | 9 | 13 |
| GO:0005783 | endoplasmic reticulum | CC | 0.0207 | 6.57E-04 | 43 | 147 |
| GO:0005835 | fatty acid synthase complex | CC | 1.03E-14 | 2.11E-17 | 25 | 5 |
| GO:0022624 | proteasome accessory complex | CC | 0.0228 | 7.36E-04 | 9 | 11 |
| GO:0044391 | ribosomal subunit | CC | 0.0088 | 2.21E-04 | 18 | 36 |
| GO:0044427 | chromosomal part | CC | 0.0407 | 0.0018 | 41 | 147 |
| GO:0044455 | mitochondrial membrane part | CC | 0.0176 | 5.49E-04 | 21 | 51 |
| GO:0046540 | U4/U6 x U5 tri-snRNP complex | CC | 0.0257 | 8.98E-04 | 6 | 4 |
| GO:0070461 | SAGA-type complex | CC | 0.0354 | 0.0014 | 7 | 7 |
| GO:0098796 | membrane protein complex | CC | 0.0052 | 1.18E-04 | 39 | 116 |
| GO:0098798 | mitochondrial protein complex | CC | 0.0291 | 0.0010 | 16 | 35 |
| GO:0000287 | magnesium ion binding | MF | 4.05E-07 | 4.53E-09 | 34 | 53 |
| GO:0003735 | structural constituent of ribosome | MF | 1.13E-07 | 1.17E-09 | 45 | 84 |
| GO:0003746 | translation elongation factor activity | MF | 0.0173 | 5.22E-04 | 10 | 13 |
| GO:0004089 | carbonate dehydratase activity | MF | 7.86E-06 | 1.05E-07 | 15 | 10 |
| GO:0004318 | enoyl-[acyl-carrier-protein] reductase (NADH) activity | MF | 2.34E-15 | 4.05E-18 | 25 | 4 |
| GO:0008080 | N-acetyltransferase activity | MF | 0.0320 | 0.0012 | 15 | 32 |
| GO:0008897 | holo-[acyl-carrier-protein] synthase activity | MF | 2.96E-14 | 6.51E-17 | 26 | 7 |
| GO:0070569 | uridylyltransferase activity | MF | 0.0025 | 5.11E-05 | 5 | 0 |

1FDR: false discovery rate

2BP: Biological Process; CC: Cellular Component; MF: Molecular Function
