# Supplementary material for: Intraspecific comparative genomics of isolates of the Norway spruce pathogen (Heterobasidion parviporum) and identification of its potential virulence factors
Source: BMC Genomics. 2018 Mar 27;19:220. doi: 10.1186/s12864-018-4610-4 (PMC5870257; doi:10.1186/s12864-018-4610-4)
Supplement: Supplementary file 21 — Table S12. Significantly over-represented GO terms of selected test gene sets against all genes in S15. (DOCX 15 kb) [file 12864_2018_4610_MOESM21_ESM.docx]

**Table S12. Significantly over-represented GO terms of selected test gene sets against all genes in S15**

| **GO IDs** | **GO annotation** | **GO**  **type1** | **FDR2** | **P-value** | **Test genes**  **set** | **Reference gene set** | **Test gene type** |
| --- | --- | --- | --- | --- | --- | --- | --- |
| GO:0005975 | carbohydrate metabolic process | BP | 1.14E-04 | 8.96E-08 | 15 | 313 | Conserved secreted |
| GO:0006032 | chitin catabolic process | BP | 2.52E-02 | 4.76E-05 | 3 | 6 | Conserved secreted |
| GO:0004553 | hydrolase activity, hydrolyzing O-glycosyl compounds | MF | 9.06E-07 | 1.43E-10 | 14 | 157 | Conserved secreted |
| GO:0030248 | cellulose binding | MF | 1.16E-03 | 1.46E-06 | 5 | 19 | Conserved secreted |
| GO:0005576 | extracellular region | CC | 1.23E-06 | 5.80E-10 | 9 | 45 | Conserved secreted |
| GO:0055114 | oxidation-reduction process | BP | 1.45E-04 | 1.15E-07 | 30 | 875 | Divergent secreted |
| GO:0042546 | cell wall biogenesis | BP | 0.01914 | 5.72E-05 | 5 | 31 | Divergent secreted |
| GO:0005975 | carbohydrate metabolic process | BP | 2.05E-04 | 2.49E-07 | 17 | 311 | Divergent secreted |
| GO:0016491 | oxidoreductase activity | MF | 1.45E-04 | 1.37E-07 | 30 | 882 | Divergent secreted |
| GO:0050660 | flavin adenine dinucleotide binding | MF | 0.03864 | 1.22E-04 | 9 | 154 | Divergent secreted |
| GO:0008810 | cellulase activity | MF | 0.01872 | 5.30E-05 | 3 | 4 | Divergent secreted |
| GO:0005199 | structural constituent of cell wall | MF | 0.00223 | 4.56E-06 | 5 | 17 | Divergent secreted |
| GO:0008236 | serine-type peptidase activity | MF | 0.00658 | 1.66E-05 | 7 | 63 | Divergent secreted |
| GO:0005507 | copper ion binding | MF | 0.00658 | 1.61E-05 | 5 | 23 | Divergent secreted |
| GO:0020037 | heme binding | MF | 0.00223 | 4.10E-06 | 12 | 190 | Divergent secreted |
| GO:0009277 | fungal-type cell wall | CC | 2.05E-04 | 2.59E-07 | 6 | 18 | Divergent secreted |
| GO:0015074 | DNA integration | BP | 0.00129 | 2.02E-07 | 7 | 16 | Duplication |

^1^FDR: false discovery rate

2BP: Biological Process; CC: Cellular Component; MF: Molecular Function
