# Supplementary material for: Intraspecific comparative genomics of isolates of the Norway spruce pathogen (Heterobasidion parviporum) and identification of its potential virulence factors
Source: BMC Genomics. 2018 Mar 27;19:220. doi: 10.1186/s12864-018-4610-4 (PMC5870257; doi:10.1186/s12864-018-4610-4)
Supplement: Supplementary file 22 — Notes. Divergent, conserved and duplicated secreted protein coding genes. (DOCX 24 kb) [file 12864_2018_4610_MOESM22_ESM.docx]

**Notes. Divergent, conserved and duplicated secreted protein coding genes.**

Combined with detected polymorphisms, highly divergent (having more than 40 variants/Kb) and conserved (having less than 5 variants/Kb without nonsynonymous SNPs and high impact variants) secreted protein coding genes were examined. The secreted proteins from protease serine families of S53 (3 genes out of 12 genes; 3/12), S28 (2/3) and S10 (2/9), and from family of cytochrome P450 (9/30) were highly divergent. Additional divergent secreted proteins include ligninolytic enzymes multicopper oxidases (5/12), manganese peroxidase (2/5), several members of PCW degrading enzymes such as GH78 (1/1), GH105 (1/3) and GH12 (2/2), and hydrophobins (4/14) based on the defined criterion. On the other hand, CAZymes from various families involved in degradation of PCW such as GH6 (1/1), GH45 (1/1), CE12 (1/1), GH10 (1/2) were found to be conserved. The most polymorphic core secreted gene encodes a hypothetic protein (evm.scaffold58.3, 124 variants/kb) and contains a FMN-binding split barrel domain (IPR012349) involved in oxidoreductase activity.

Laccase, a member of multicopper oxidase family exhibited polymorphism in *H. parviporum*. Fungal laccases have been reported to have broad substrate specificity and perform various physiological functions including oxidation of phenolic substrates (e.g. lignin), pigment production, sporulation and infection of plant hosts as virulence factors [1]. Generally, fungal laccases possess four conserved motifs (L1 to L4) distributed throughout the sequence that coordinate the copper ion as a chelator in the center of the enzyme [2]. In particular, histidines associated with copper binding were housed in those motifs. The axial coordination that influences the redox potentials (E0) which further correlate with the enzyme activity (leucine or phenylalanine correspond to higher E0 and methionine corresponds to lower E0) was located at the tenth position downstream the preserved cysteine residue in L4 [1, 2]. Detailed examination of the secreted laccases showed that all those important motifs and residues (His, Cys, and Phe) were highly conserved in *H. parviporum* despite a larger number of variants in other parts of genes. Basically, all isolates share the same number of secreted multicopper oxidase except for a putative partial deletion in one copy of this enzyme in S8 (Fig 5). Interestingly, another copy of laccase (evm.scaffold67.10) was situated within an outlier window of negative *D* value (Table 4), indication of being under positive selection and simultaneously possessing two nonsynonymous SNPs conferred by isolate S12 (Table 5). Although the two nonsynonymous SNPs did not affect its conserved motifs, it is tempting to divert some attention to it for future study.

Overrepresentation of genes relevant to DNA integration in both duplicated and novel gene sets highlights the predominance and plasticity of TEs. Apart from one copy of cytochrome P450, four other secreted proteins were also found duplicated. They were one catalase (evm.scaffold59.8) and one CBM50 (evm.scaffold38.58; LysM domain) duplicated in S7, one hypothetic protein (evm.scaffold34.57; containing CFEM domain) duplicated in S12 and one hypothetic protein (evm.scaffold67.32; containing non-heme dioxygenase N-terminal domain) duplicated in S2. One secreted protein duplicated in the deletion gene set was likely to be gene prediction artifact as no homologs in the database could be found.

CFEM domain is a cysteine-rich extracellular membrane domain and shown to appear more frequently in pathogenic fungi than non-pathogenic fungi [3, 4]. Gene duplication and tandem duplication have been proposed as possible mechanisms for the expansion of fungal CFEM domain [4]. One gene containing CFEM domain found duplicated in *H. parviporum* S12 and other three CFEM domain-containing genes (evm.scaffold7.65, evm.scaffold7.69 and evm.scaffold7.70) arranged in scaffold7 in tandem manner further bolster the mentioned mechanisms. Nevertheless, its putative duplication in the least virulent isolate merely obscures the functional connection of this domain to the fungal virulence considering its proposed role in pathogenesis in other fungi [3, 4]. As non-pathogenic fungi can also contain CFEM domain such as in *Saccharomyces cerevisiae* functioning in biogenesis and maintaining the integrity of cell wall [5], the CFEM-containing proteins in *H. parviporum* could be involved in more yet to be characterized processes.

**References**

1. Moreno LF, Feng PY, Weiss VA, Vicente VA, Stielow JB, de Hoog S. Phylogenomic analyses reveal the diversity of laccase-coding genes in Fonsecaea genomes. Plos One. 2017;12(2).

2. Kumar SVS, Phale PS, Durani S, Wangikar PP. Combined sequence and structure analysis of the fungal laccase family. Biotechnol Bioeng. 2003;83(4):386-94.

3. Kulkarni RD, Kelkar HS, Dean RA. An eight-cysteine-containing CFEM domain unique to a group of fungal membrane proteins. Trends Biochem Sci. 2003;28(3):118-21.

4. Zhang ZN, Wu QY, Zhang GZ, Zhu YY, Murphy RW, Liu Z, et al. Systematic analyses reveal uniqueness and origin of the CFEM domain in fungi. Sci Rep. 2015;5:13032.

5. Mrsa V, Ecker M, Strahl-Bolsinger S, Nimtz M, Lehle L, Tanner W. Deletion of new covalently linked cell wall glycoproteins alters the electrophoretic mobility of phosphorylated wall components of Saccharomyces cerevisiae. J Bacteriol. 1999;181(10):3076-86.
